# Partial correlation network analysis identifies coordinated gene expression within a regional cluster of COPD genome-wide association signals

**DOI:** 10.1371/journal.pcbi.1011079

**Published:** 2024-10-17

**Authors:** Michele Gentili, Kimberly Glass, Enrico Maiorino, Brian D. Hobbs, Zhonghui Xu, Peter J. Castaldi, Michael H. Cho, Craig P. Hersh, Dandi Qiao, Jarrett D. Morrow, Vincent J. Carey, John Platig, Edwin K. Silverman

**Affiliations:** 1 Channing Division of Network Medicine, Brigham and Women’s Hospital, Boston, Massachusetts, United States of America; 2 Harvard Medical School, Boston, Massachusetts, United States of America; 3 Harvard T.H. Chan School of Public Health, Boston, Massachusetts, United States of America; 4 Division of Pulmonary and Critical Care Medicine, Brigham and Women’s Hospital, Boston, Massachusetts, United States of America; 5 Division of General Internal Medicine and Primary Care, Brigham and Women’s Hospital, Boston, Massachusetts, United States of America; 6 Department of Genome Sciences, University of Virginia, Charlottesville, Virginia, United States of America; 7 Department of Biomedical Engineering, University of Virginia, Charlottesville, Virginia, United States of America; ., CANADA

## Abstract

Chronic obstructive pulmonary disease (COPD) is a complex disease influenced by well-established environmental exposures (most notably, cigarette smoking) and incompletely defined genetic factors. The chromosome 4q region harbors multiple genetic risk loci for COPD, including signals near *HHIP*, *FAM13A*, *GSTCD*, *TET2*, and *BTC*. Leveraging RNA-Seq data from lung tissue in COPD cases and controls, we estimated the co-expression network for genes in the 4q region bounded by *HHIP* and *BTC* (~70MB), through partial correlations informed by protein-protein interactions. We identified several co-expressed gene pairs based on partial correlations, including *NPNT-HHIP*, *BTC*-*NPNT* and *FAM13A*-*TET2*, which were replicated in independent lung tissue cohorts. Upon clustering the co-expression network, we observed that four genes previously associated to COPD: *BTC*, *HHIP*, *NPNT* and *PPM1K* appeared in the same network community. Finally, we discovered a sub-network of genes differentially co-expressed between COPD vs controls (including *FAM13A*, *PPA2*, *PPM1K* and *TET2)*. Many of these genes were previously implicated in cell-based knock-out experiments, including the knocking out of *SPP1* which belongs to the same genomic region and could be a potential local key regulatory gene. These analyses identify chromosome 4q as a region enriched for COPD genetic susceptibility and differential co-expression.

## Author summary

Complex diseases, such as chronic obstructive pulmonary disease (COPD), are characterized by multifactorial causes, including multiple genetic variants and a variety of molecular functions. A 70 megabase genomic region on chromosome 4 harbors some of the strongest genetic associations to COPD based on genome-wide association studies (GWAS). In this work we study the co-expression patterns of genes located in this genomic region. We developed a new approach to compute partial correlations between pairs of expressed genes, using prior information regarding the mediating genes from protein-protein interaction networks. We identified potential biological connections between several of the COPD-related GWAS genes in this region, including *BTC*, *NPNT*, *PPM1K* and *HHIP*, suggesting possible functional co-regulation. Furthermore, by comparing COPD cases and control subjects, we found multiple network edges whose co-expression changed between health and disease, particularly the edge between *CXCL10* and *CXCL11*. Some of these genes were previously implicated in cell-based knock-out experiments, suggesting a common regulator, namely *SPP1*. These analyses provide insight into regional gene regulation of GWAS genes that may be related to COPD pathogenesis.

## 1. Introduction

Genome-wide association studies (GWAS) have identified thousands of genomic regions associated with complex diseases, including Chronic Obstructive Pulmonary Disease (COPD). These variants overwhelmingly lie in non-coding genomic regions and likely influence gene regulation. Such regulatory effects are typically classified as cis-acting local effects and trans-acting distant effects [1]. Cis-acting effects, such as enhancers, usually occur within topological associated domains (TAD) that are less than one megabase in size [2]. Trans-acting effects, such as transcription factors, can act anywhere in the genome where appropriate DNA binding sites are located. Collectively, these regulatory effects can lead to coordinated changes in gene expression levels. Furthermore, functionally related groups of genes have been shown to exhibit correlated expression [3–5], suggesting that the inverse is also true: genes with correlated expression are functionally related and potentially co-regulated. Thus, using results from a large, collaborative COPD GWAS study [6], we wanted to investigate whether a dense collection of COPD-associated loci on chromosome 4q also contained disease relevant co-expression signals.

**Fig 1:**
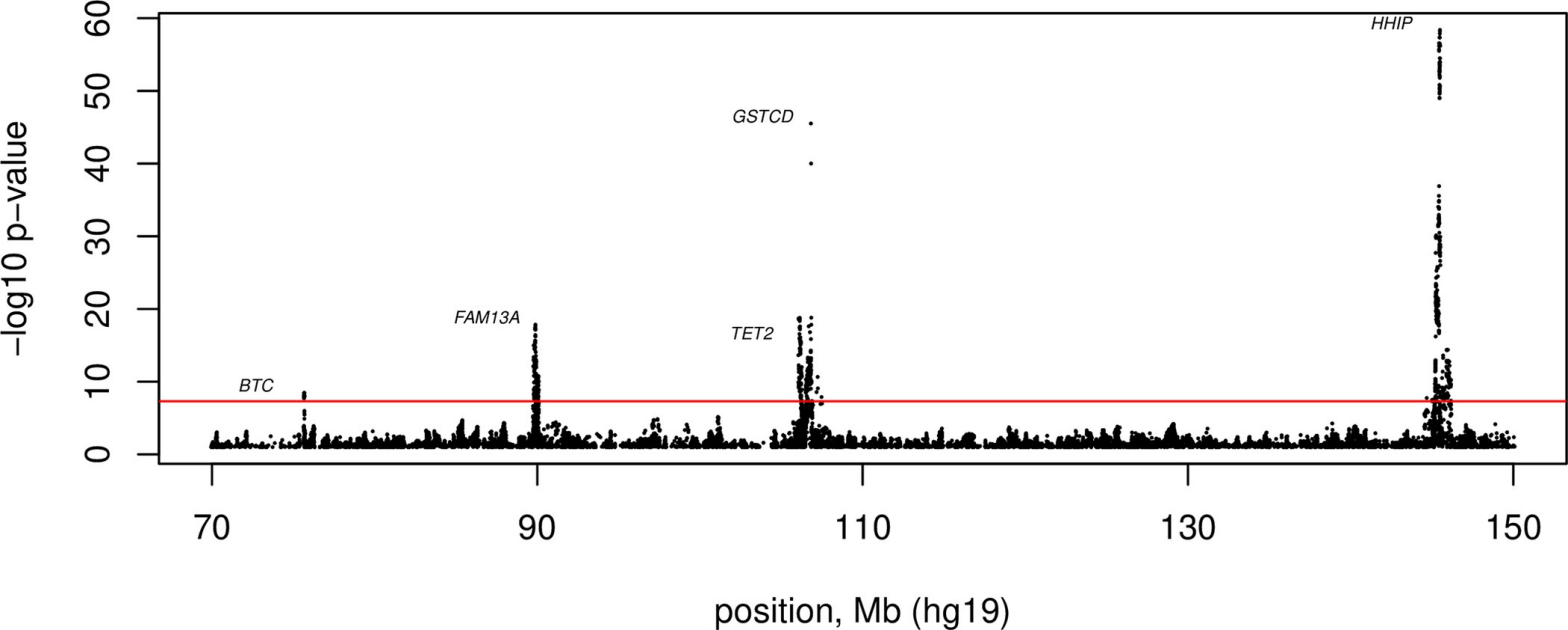
Regional association plot of the chromosome 4q genomic region bounded by COPD risk loci near *BTC* and *HHIP*. Regional association plot of the 4q genomic region bounded by COPD risk loci near *BTC* and *HHIP* from the Sakornsakolpat et al. 2019 COPD GWAS study [6]. *P*-values are two-sided based on Wald statistics (35,735 cases and 222,076 controls). The loci are labeled according to the gene with a transcription start site closest to the top GWAS variant at each locus. The red line corresponds to genome-wide significance (p = 5 x 10^−8^).

Five loci associated with COPD are located on chromosome 4q within a 70 Mb region bounded by the *BTC* and *HHIP* genes **(Fig 1)**, with genome-wide significant associations near *HHIP*, *FAM13A*, *GSTCD*, *TET2* and *BTC*. The first two genome-wide significant COPD GWAS loci were identified on chromosome 4q (near *HHIP*) and chromosome 15q [7,8]. In 2010, a third COPD GWAS locus in the *FAM13A* gene was also found on chromosome 4q [9]. The International COPD Genetics Consortium (ICGC) identified 22 genome-wide significant loci for COPD; four of these loci were located on chromosome 4q [10]. When the ICGC and UK Biobank were combined in a GWAS meta-analysis of COPD, more than 80 genome-wide significant regions were found, including an additional COPD GWAS locus on chromosome 4q near *BTC* [6]. Finding five loci on chromosome 4q raised the question whether this region is statistically enriched or whether this co-occurrence is due to chance. Furthermore, it is unknown whether there is any shared regulation between these regional genes.

Identifying the functional gene or genes within each complex disease GWAS locus is challenging. For several genes in chromosome 4q COPD GWAS loci, including *HHIP* [11], *FAM13A* [9], and *TET2* [12], animal models and other functional evidence have strongly supported their role in COPD pathogenesis. Other genes near chromosome 4q COPD GWAS loci in the *CXCL* [13] family, *NPNT* [14], and *PPA2*, have some biological support [6], although the links between the associated genetic variants and the causal gene have not been definitively established.

Previous attempts have been made to leverage gene expression profiles in COPD to understand the association between GWAS variants and regional genes. Using transcriptomic data from lung tissue, Morrow et al. 2017 [15] showed that genes near COPD GWAS loci were not differentially expressed in COPD cases and controls; however, putative molecular interactors with those genes were differentially expressed. Later, Paci et al. [16] found three network modules in a COPD co-expression network; one of these modules included a COPD GWAS gene (*AGER)* and a COPD lung protein biomarker *CAVIN1* [17]as highly representative genes, supporting the hypothesis of coordinated changes to gene expression levels in COPD patients. However, the standard gene correlation networks used to estimate co-expression only consider pairwise relationships between genes. In biological networks, a pair of genes with a weak marginal (direct) relationship may have an indirect relationship mediated by other genes. Partial correlations provide an effective estimate of conditional co-expression between two genes [18–20], with a schematic example of partial correlations provided in **S1 Fig.** Furthermore, it is likely that the effects of genetic variants in complex diseases cannot be captured by a single type of omics data, and including different biological data sources may facilitate the removal of indirect effects, allowing the identification of correlations that would not be detected otherwise. For example, a non-negative matrix factorization approach [21] constrained gene co-expression to a protein-protein interaction (PPI) network to stratify COPD patients. Others [22] have prioritized COPD candidate genes through a weighted COPD PPI network using expression information. Finally, multi-tissue transcriptomic network approaches [23] have been used to compare gene expression profiles between COPD patients.

**Fig 2:**
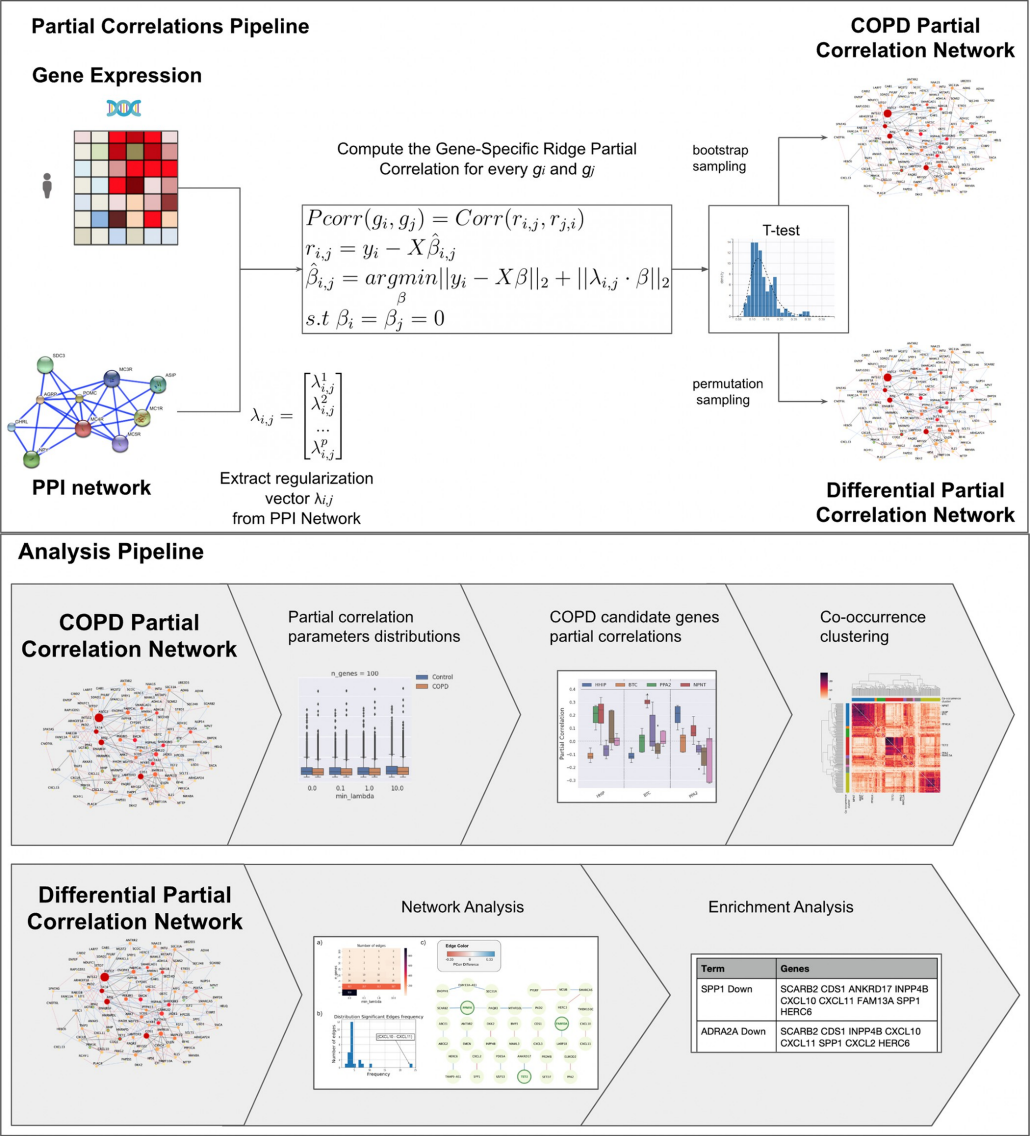
Graphical Abstract. The first box shows how the COPD and the Differential partial correlation networks have been built. Given a source of biological information, such as a PPI network or set of functional annotations, for every gene i and j (g_i_,g_j_) we extract the gene specific regularization vector *λ*_*i*,*j*_, that contains the distance between gene g_i_ and g_j_ to all of the other genes, in our case the inverse of the personalized PageRank algorithm (more details in **S1 Text**). For every pair of genes (g_i_,g_j_), we compute the Gene-Specific Ridge Partial Correlation. Finally, to build the partial correlation network we run a t-test, using permutation and bootstrap procedures, respectively. The second box contains the analysis done on the two networks from (1) the COPD subjects and (2) a differential network of COPD patients vs Healthy controls. Both analyses provide insights into the presence of a potential co-regulatory network in this genomic region.

In this work, we leveraged co-expression networks of genes in the chromosome 4q COPD risk region through partial correlations of transcriptomic data, using protein-protein interaction (PPI) networks as a constraint, to estimate only marginal (direct) relationships between genes. The PPI provided an educated guess regarding the mediating genes for any given pair of genes. In particular, we found: 1) The genes in the 4q COPD risk region are part of a dense co-expression network, leading to the discovery of multiple co-expression links between COPD GWAS candidate genes (CCG) (Section 2.2); 2) a network module of co-expressed genes including *HHIP*, *BTC*, *NPNT* and *PPM1K* (Section 2.3); 3) the existence of a differential co-regulatory sub-network between individuals with COPD and healthy controls, including *FAM13A*, *PPA2*, *PPM1K* and *TET2*. Multiple genes of this sub-network were previously associated in cell-based knock-out experiments, including *SPP1* (Section 2.4). **Fig 2** provides a graphical abstract of our approach.

## 2. Results

### 2.1. COPD GWAS Signals and Study Population

Sakornsakolpat et al. [6] and Hobbs et al. [10] identified a set of 83 GWAS loci associated to COPD, and we evaluated whether these loci significantly clustered within small regions of the genome or they were scattered randomly across the genome; see **Methods** for details. We observed that altogether the COPD GWAS regions were more clustered than expected. In particular, the overall number of regions with 3 COPD GWAS hits (**S5 Fig**) within 34.28MB (n = 26), was statistically significant (p = 0.006) compared to 100,000 simulations, see **S5 Table** for these regions.

We further analyzed the clusters of GWAS loci focusing on chromosome 4q, since two of the most well-established COPD GWAS susceptibility loci (*FAM13A* and *HHIP*) are located in this region. There are two overlapping clustered regions of triplet GWAS signals in chromosome 4q between rs4585380 and rs34712979 based on our simulations. The *HHIP* GWAS lead SNP is only 38.7 Mb away from rs34712979; if we merge in *HHIP*, we obtain the co-occurrence of five COPD GWAS signals within 70 Mb. This made us speculate that there could be a network of interactions between these regional genes that would be relevant for COPD pathogenesis. Thus, we decided to investigate COPD-relevant gene co-expression networks within this region.

We used a study population with RNA-Seq data generated in lung tissue samples from 458 COPD cases and 329 control subjects in the Lung Tissue Research Consortium (LTRC); **Table 1** shows the clinical characteristics of these subjects. We focused exclusively on 224 genes expressed within the *4q COPD risk region* (see **Methods**). This included eight *COPD candidate genes (CCG)* based on *OpenTargets* [24] analysis of top COPD GWAS SNPs or previous identification as likely functional COPD genes in Sakornsakolpat et al [6] based on having at least two sources of supporting evidence (detailed information is listed in **S1 Table**). Chromosome 4q CCG genes include: *BTC*, *FAM13A*, *NAP1L5*, *PPM1K*, *NPNT*, *PPA2*, *HHIP* and *TET2*; *EPGN* was a regional candidate gene that was not included because it had low expression in the LTRC dataset.

**Table 1:** LTRC Subject Characteristics. Values represent mean and (standard deviation) for continuous variables and count (percentage) for binary variables. The values with (*) are statistically different between cases and controls, p-value <10^−3^. 34 control patients and 28 case patients have missing data for smoking status.

	Control Subjects (n = 329)	COPD Cases (n = 458)
**Age (years)**	61.8 (12.3)	63.2 (9.3)
**Body Mass Index**	29.0 (6.0)	26.4 (5.3)*
**FEV1 (%predicted)**	96.3 (12.6)	42.0 (20.2)*
**FEV1/FVC**	0.77 (0.06	0.45 (0.15)*
**Smoking (Pack Years)**	19.8 (27.4)	46.9 (32.2)*
**Time since quitting ex-smokers (years)**	18.5 (14.5)	8.6 (9.7)*
**Gender (male)**	128 (38.9%)	249 (54.4%)*
**Race non-White**	21 (6.4%)	28 (6.1%)
**Race white**	308 (93.6%)	430 (93.9%)
**Smoking Ever**	203 (67.2%)	409 (94.9%)*
**Smoking Former**	187 (61.9%)	378 (87.7%)*
**Smoking Current**	16 (5.3%)	31 (7.2%)
**GOLD Spirometry Grade**		
**0.0**	329 (100.0)	0(0)*
**2.0**	0(0)	208 (45.4) *
**3.0**	0(0)	89 (19.4) *
**4.0**	0(0)	161 (35.2) *

### 2.2 Significant Partial Correlations between Multiple COPD Candidate Genes

**Fig 3:**
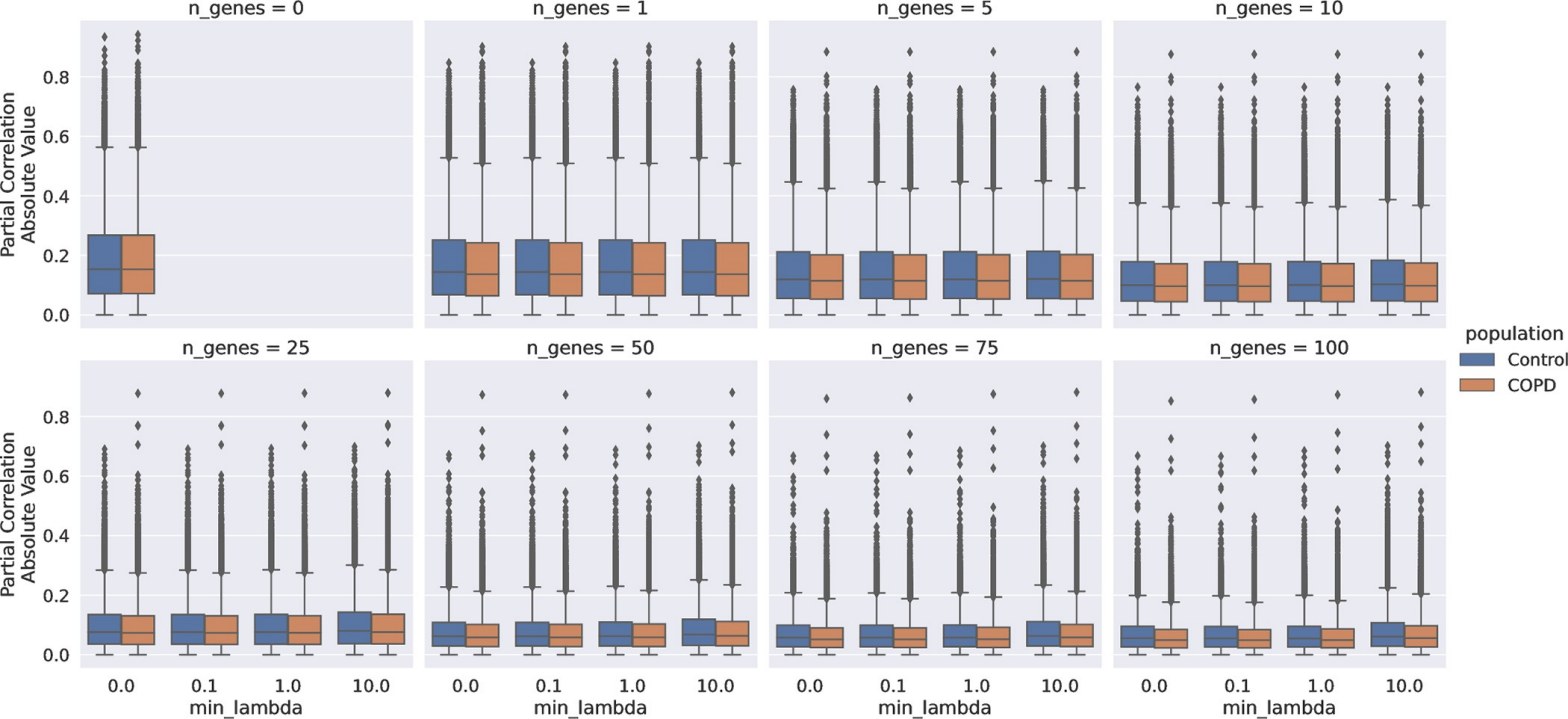
Absolute Partial Correlation Distribution. Distributions of the absolute value of the partial correlations between genes in the *4q COPD risk region* using the LTRC RNA-seq data, with different parameter settings, including *min_lambda* (magnitude of the regularization parameter) and *n_genes* (number of controlling genes).

To assess the marginal (direct) relationships between genes in the chromosome 4q region, we computed the partial correlation network using RNA-seq data from lung tissue samples in 458 COPD cases and 329 control subjects from the LTRC cohort. We leveraged protein-protein interaction data [25] (STRING PPI, see **Methods)** together with the LTRC gene expression data to compute the region-specific partial correlation score between 224 genes within the 4q COPD risk region (see **Methods**). We computed the partial correlation coefficients choosing different values for the two parameters defined in **Section 4.2**: the number of controlling genes (*n_genes*) for the partial correlation and the magnitude of their regularization (*min_lambda*) in the ridge regression, *i*.*e*., the minimum value of the vector lambda. In general, the higher *n_genes*, or the lower *min_lambda*, the more direct the co-expression of two genes will be when assessed by the partial correlation. Since it would not be feasible to test all possible parameter combinations, we selected a reasonable set of values that would explore different situations. For *n_genes* we evaluated [0, 1, 5, 10, 25, 50, 75, 100] and for *min_lambda* we evaluated [0, 0.1, 1, 10]. Note that when *n_genes* is equal to 0, the partial correlation is the same as the correlation (regardless of the value of *min_lambda*), and when *min_lambda* is 0, then it is equal to the canonical partial correlation (*i*.*e*., without the regularization factor), thus we have a total of 29 combinations, because when *n_genes* is 0 we consider only the values of *min_lambda* equal to 0, as shown in **Fig 3**. As expected, the distribution of the partial correlations shrunk when increasing the number of controlling genes as well as when the regularization factor (*min*_*lambda)* is smaller.

**Fig 4:**
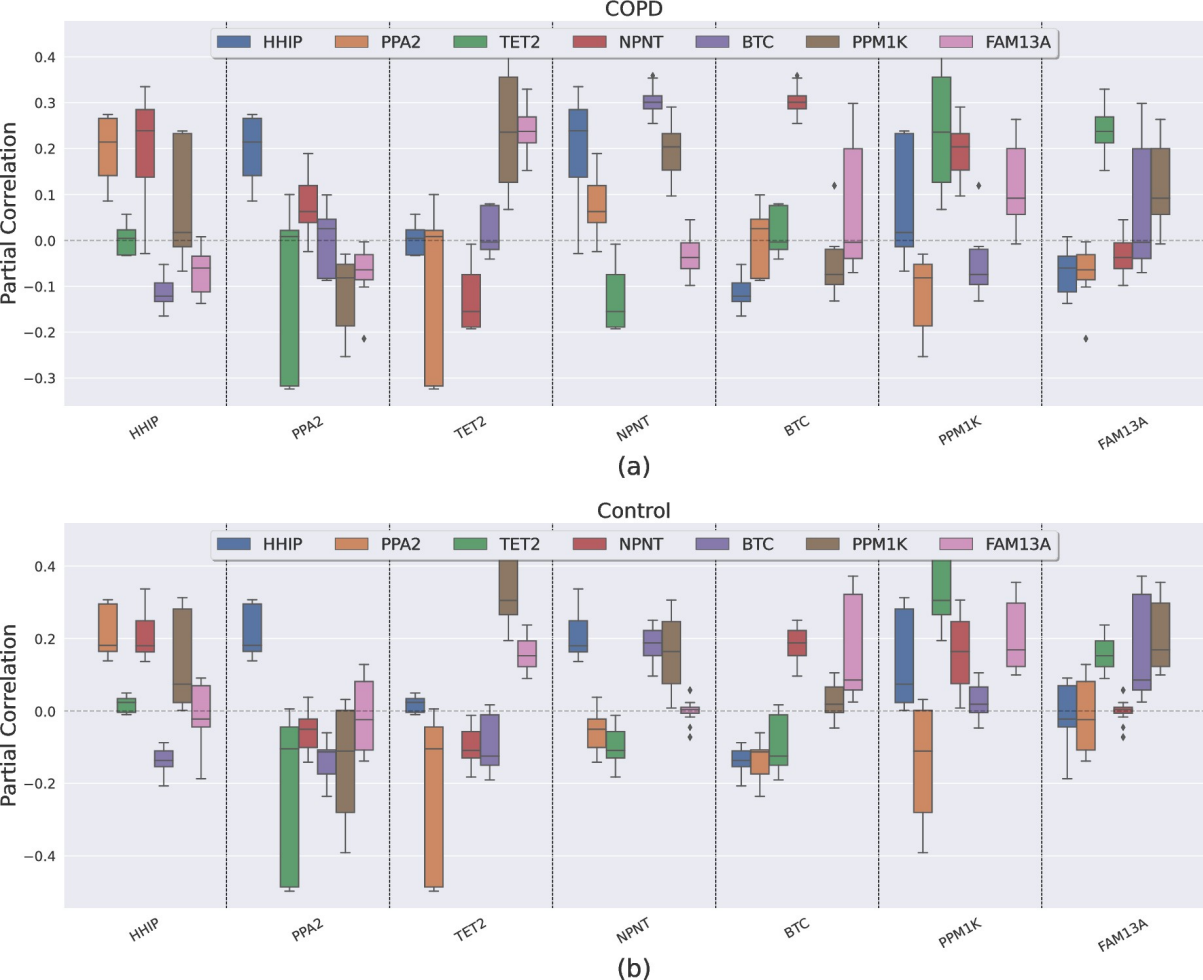
COPD Candidate Gene (*CCG)* partial correlation distributions. Boxplot of *CCG* partial correlation distribution over the 29 different parameter combinations in (a) COPD case and (b) control subjects. *NAP1L5* has been removed because it did not show any significant partial correlation. A gene pair is identified by the position on the x-axis and the color of the distribution.

The Partial Correlation distributions (**Fig 3**) highlight strong partial correlations in the top quartile of the distributions that are present regardless of the regularization factor, which indicate the presence of pairs of genes with strong marginal (direct) relationships. Looking specifically for relationships between the COPD GWAS risk loci, we focused on the partial correlations among CCG. **Fig 4** shows boxplots of the partial correlation distributions across the 29 different parameter values for CCG. A pair of genes can substantially vary in their partial correlation depending on the selected parameter values. **S2 Table** shows the median values of the distributions of the partial correlations. However, especially for the COPD population, we noted that some interesting pairs of genes always had a partial correlation much greater (or less) than 0 regardless of the choice of the algorithm parameters. Furthermore, for *HHIP-NPNT*, *HHIP-PPA2*, *BTC-NPNT*, and *FAM13A-TET2*, we obtained similar results in at least two of three independent cohorts using previously reported and publicly available lung tissue gene expression assessments [26]: GSE23352 (Laval), GSE23545 (Groningen), and GSE23529 (University of British Columbia) (**S2 Fig**). Of interest, these consistent partial correlations were not identified between nearby genes in the same COPD GWAS locus (e.g., FAM13A-PPM1K, NPNT-PPA2), but rather represented relationships between genes located at distances greater than typical topological associated domains (1 MB). In addition to these replicated partial correlations, there were several partial correlations only observed in the LTRC population, including *HHIP*-*BTC*, *NPNT*-*PPM1K*, *NPNT*-*TET2*, *FAM13A*-*PPM1K* and *PPM1K*-*TET2*.

Since gene co-expression algorithms could be strongly affected by other phenotypic variables (e.g., age, sex, race, FEV1), and differences in cell type composition between the case and control groups, we have tested whether the analysis would benefit from adding such variables in the model. While adding phenotypic variables does not affect the Partial Correlation score, cell composition does affect the partial correlation; however, the effect is mitigated with the increasing number of controlling genes (**S3 Fig**).

Furthermore, to verify the stability of our results to PPI study bias or lack of condition-specificity in the edges [27], we compared the partial correlation in two randomization settings: a randomized PPI and a randomized Prior, see **Methods**. As shown in **S4 Fig**, the randomization of the PPI has a similar effect (0.08 as mean absolute difference) to the randomization of the Prior (0.1), *i*.*e*. not providing any biological prior to the algorithm. Therefore, we can suggest that the partial correlation algorithm is not strongly affected by possible inaccuracies in the PPI such as study bias or lack of condition-specificity in the edges, and that the PPI does carry relevant biological information, specifically in its topology and not only in its degree distribution.

### 2.3 *NPNT*, *PPM1K*, *BTC*, and *HHIP* co-occur in the same module in COPD partial correlation networks

**Fig 5:**
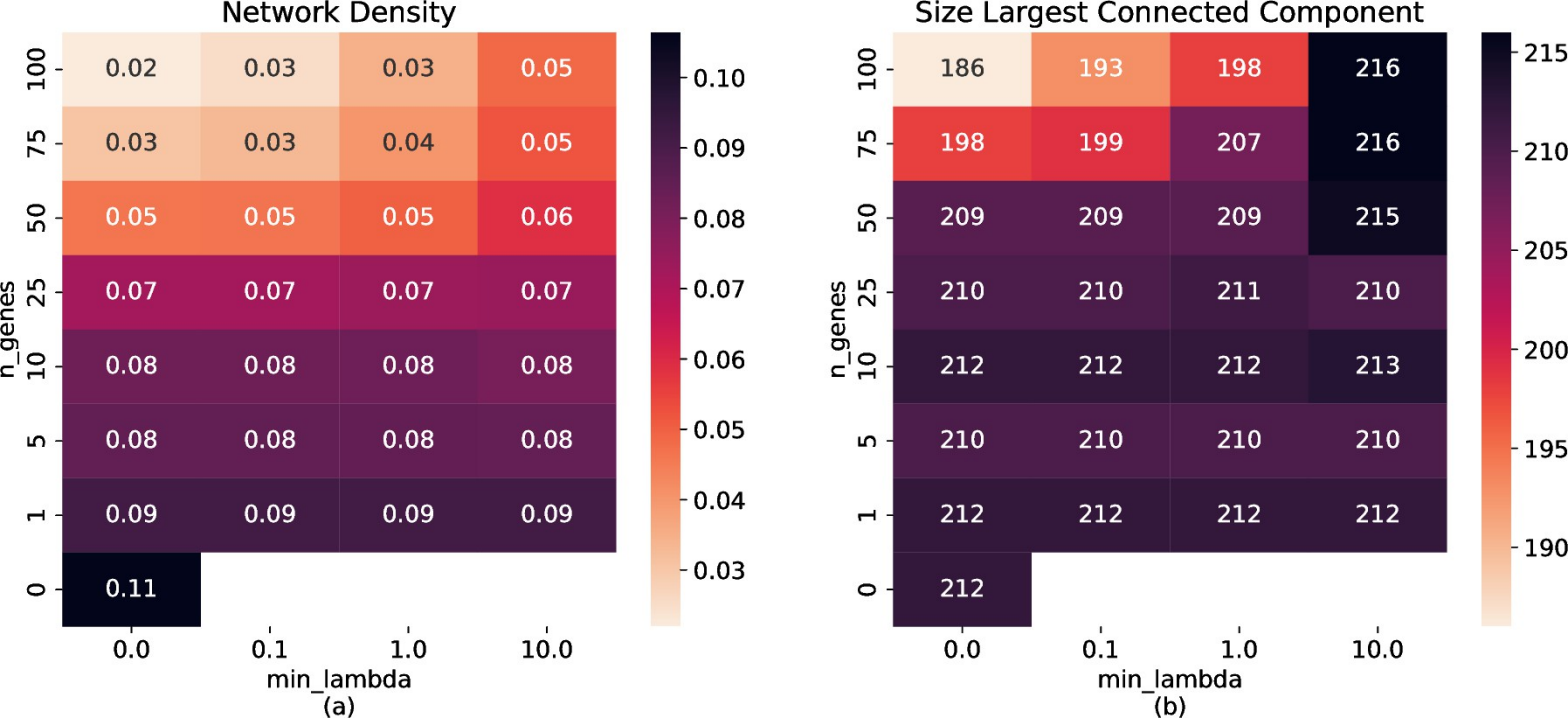
Partial Correlation Network topologies. (a) shows the network density (fraction of all possible gene pairs that have an edge) and (b) shows the size of the largest connected component (the highest number of nodes that are connected to each other directly or through other nodes). Each network is identified by the set of parameters used to compute the Partial Correlation (*n_genes*, *min_lambda*). *n_genes* is the number of controlling genes and *min_lambda* is the normalization parameter, which are used to compute the partial correlation.

We assessed the topological relatedness of genes in the 4q COPD risk region (224 genes), through a consensus network approach. For each set of model parameters in Section 2.2, we built a network using the 224 genes as nodes and significant partial correlations (FDR <0.01) between genes as unweighted edges. The significance of the partial correlation was computed through a bootstrap approach, see details in the **Methods** section. We obtained 29 Partial Correlation networks, one for each set of parameters. **Fig 5** provides details of their topology including network density and size of the largest connected component. Concordant with our previous results (Section 2.2), while the correlation network is very dense, some combinations of the partial correlation networks yield a sparser network with an equal or larger connected component. There is a continuous space from correlation to partial correlations: the greater the number of controlling nodes or the smaller the regularization, the sparser the resulting network.

**Fig 6:**
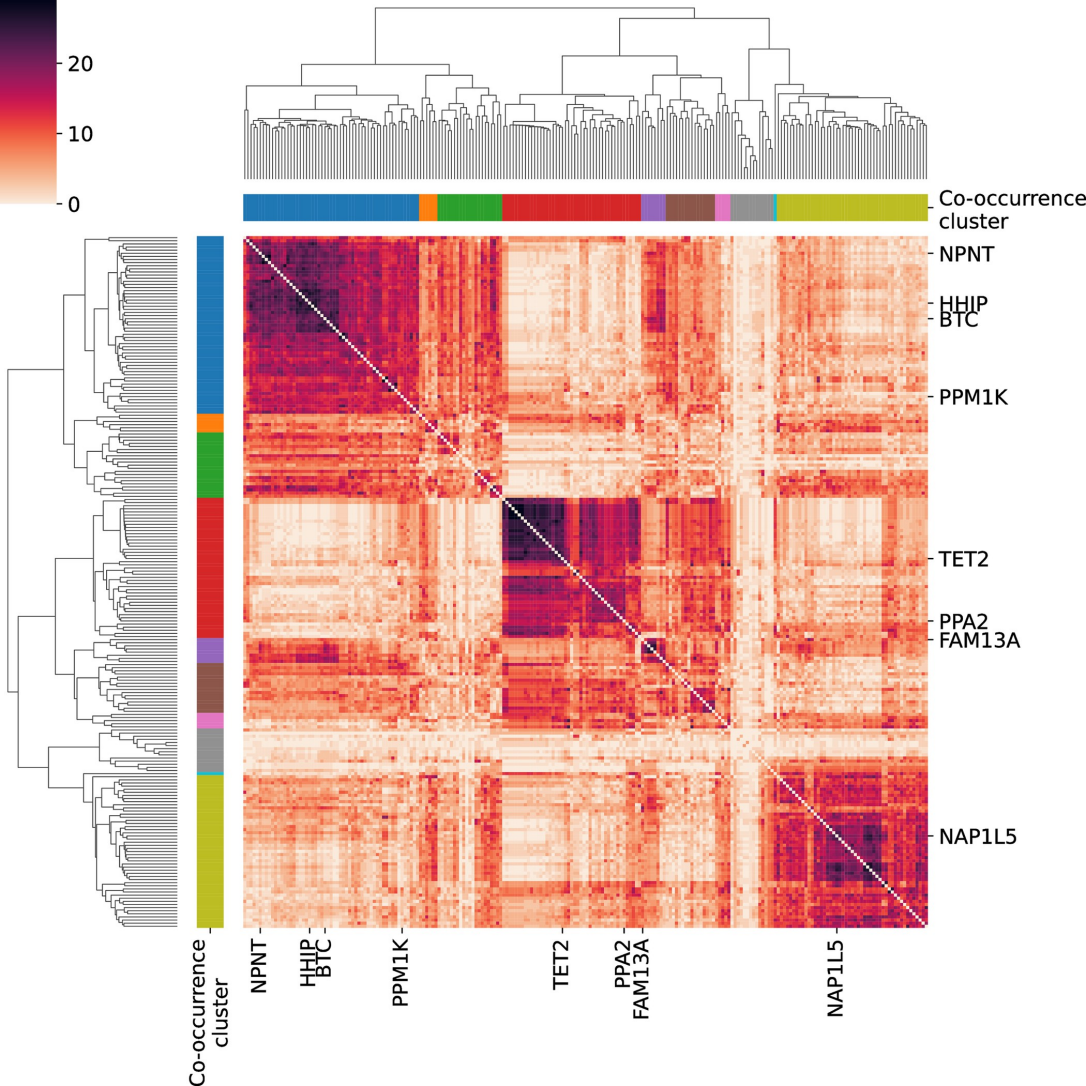
Partial Correlation Co-occurrence clustering heatmap. Each entry of the matrix represents how many times two genes appear in the same module across partial correlation networks with different parameter settings. Hierarchical clustering of genes based on their co-occurrence across the partial correlation networks identified 10 clusters. *CCG* are shown on the two axes.

To determine whether pairs of CCG are involved in close co-regulatory subnetworks, in each of these 29 networks, we partition (cluster) genes into groups that were densely connected, *i*.*e*. network modules, using a modularity maximization algorithm (see **Methods**). Out of these 29 partitions (clusterings), we obtained the co-occurrence matrix (**Fig 6**), *i*.*e*., the number of times two genes appear in the same module in the 29 networks. Finally, to have a partition of the genes robust to the different algorithm parameters, we used hierarchical clustering to group genes with high co-occurrence across the 29 networks generated using different model parameters. The genes in the 4q COPD risk region are often present in similar modules throughout the networks (**S3 Table**). Interestingly, *HHIP*, *NPNT*, *BTC*, and *PPM1K* tend to occur in the same module; genes in this module have an average co-occurrence of 57%. Another module, containing *PPA2* and *TET2*, also has an average co-occurrence of 57%. We note that although *FAM13A* and *TET2* had a high partial correlation distribution (Section 2.2), they do not usually belong to the same network module (average co-occurrence of 31%). In a gene set enrichment analysis we found *HHIP*, *NPNT*, *BTC*, and *PPM1K* to be enriched for histone modifications in various cell types relevant in COPD patients, *i*.*e*. monocytes, fibroblasts, lymphocytes, and alveolar basal epithelial cells (**S4 Table**).

Although we have focused on the chromosome 4q cluster of COPD GWAS signals, we also investigated another COPD GWAS cluster on chromosome 5q between variants rs62375246 and rs13140176, since it harbors five COPD GWAS hits within a 38MB area. Four of the five genes linked to these variants, assigned in the paper Sakornsakolpat et al. 2019 [6] as closest genes to the variant, were present in the LTRC lung tissue gene expression data: *HSPA4*, *CCDC69*, *ADAM19*, and *FGF18*. **S6 Fig** shows the partial correlation results obtained by this analysis. *FGF18* is differentially partially correlated to *CCDC69* in Cases vs Controls. Furthermore, we identified a cluster containing three of these four CCGs (*HSPA4*, *ADAM19*, and *FGF18*). In a GSEA analysis, we found that the cluster containing these three genes was enriched for Monocyte-derived alveolar infiltration [28].

### 2.4 Differential partial correlations enriched for down-regulated genes in cell-based knock-out experiments

**Fig 7:**
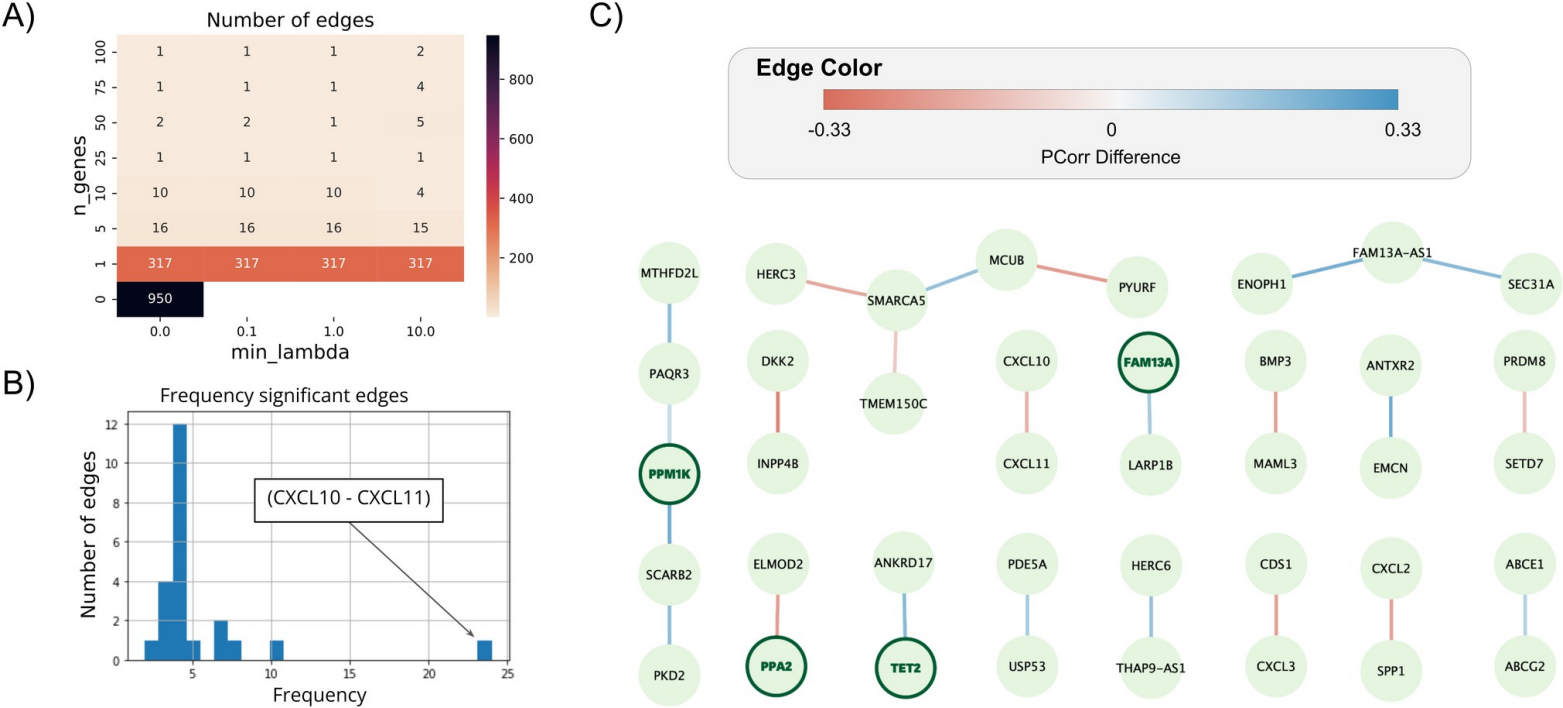
COPD Candidate Genes are present in the differential partial correlation network. An edge between two genes is present if the two genes are significantly differentially partially correlated (FDR<0.1). (a) The number of significant edges obtained for each set of parameters. (b) The histogram showing how frequently an edge was significant in the partial correlation networks estimated using different parameters. The edge with the highest frequency is (*CXCL10*-*CXCL11*). (c) The network obtained by edges present in the partial correlation with >1 controlling genes (*n_genes*). The edge color is the average partial correlation difference obtained with all of the examined parameters. Blue edges represent pairs of genes that are more positively correlated in the control subjects, red edges represent pairs of genes that are more correlated in COPD subjects. COPD Candidate Genes (CCG) are highlighted with a dark green outline.

To identify coordinated network changes among genes in the chromosome 4q COPD risk regions, we analyzed differential edges, *i*.*e*., gene pairs whose partial correlation is significantly different between the control population (329 non-COPD subjects) versus the case population (458 COPD subjects) based on permutation analysis (n = 1000). We carried out the analysis for each set of parameters (29) of the partial correlation algorithm, and then we extracted edges that were significantly different between cases and controls for a given set of parameters (FDR ≤ 0.1). **Fig 7A** shows the number of edges obtained for each parameter combination. Given the significant differences in the number of edges of each network, we selected only edges that were obtained in partial correlation networks with 5 or more controlling genes, obtaining 23 distinct edges connecting 39 nodes (**Fig 7C**). The edge frequency, *i*.*e*., how many times an edge was significant in the selected partial correlation networks, is shown in **Fig 7B**. Interestingly, four of the eight CCG had at least one differential edge, including: *PPM1K*, *PPA2*, *FAM13A* and *TET2*. Another interesting set of genes that is present in the differential network is a group of chemokines: *CXCL2*, *CXCL3*, *CXCL10* and *CXCL11*, which have been implicated in COPD pathophysiology [13]. Finally, multiple genes in this region have been discovered in cell-based knock-out experiments from the LINCS L1000 project [29] (**Table 2**), which provide supportive evidence for the presence of a co-regulatory network in the chromosome 4q COPD risk region. Of particular interest, when the 4q gene *SPP1* is knocked out, eight other genes in the 4q region are also affected, including *FAM13A*. Furthermore, *SPP1* acts as a cytokine involved in enhancing production of interferon-gamma and interleukin-12 and reducing production of interleukin-10 and is essential in the pathway that leads to type I immunity [30].

**Table 2:** Genes in the differential partial correlation network are involved in cell-based knock-out experiments. The table shows the enrichment analysis of the nodes of the Differential Partial Correlation Network, COPD vs healthy controls, with the LINCS L1000 project perturbation gene sets. The first column shows the name of the knockout experiment, then the p-values and the adjusted p-values for multiple hypothesis testing. Finally, the gene column highlights the genes there are present in the differential network and in the knock-out gene set.

Knockout experiment	P-value	Adjusted P-value	Genes
SPP1 Down	8.55E-10	5.66E-06	SCARB2 CDS1 ANKRD17 INPP4B CXCL10 CXCL11 FAM13A SPP1 HERC6
ADRA2A Down	1.88E-08	5.02E-05	SCARB2 CDS1 INPP4B CXCL10 CXCL11 SPP1 CXCL2 HERC6
HPD Down	2.27E-08	5.02E-05	INPP4B CXCL11 MCUB SPP1 CXCL3 CXCL2 ABCG2 HERC6
AKR1C3 Down	4.41E-07	4.88E-04	INPP4B CXCL10 CXCL11 CXCL3 CXCL2 ABCG2 HERC6
OSCAR Up	3.64E-07	4.88E-04	SCARB2 CXCL10 CXCL11 CXCL3 CXCL2 ABCG2 HERC6

## 3. Discussion

In previous genome-wide association studies [6] more than 80 genomic regions associated with COPD have been identified. We identified non-random clustering of GWAS signals in multiple genomic regions. Although there is still not a functional explanation, multiple COPD GWAS signals have been identified within a broad genomic region (~70 Mb) on chromosome 4q, within the region bounded by the *BTC* and *HHIP* genes. Leveraging Partial Correlation to focus the analysis on smaller regions of the genome (specifically in chromosome 4q) has provided interesting insights into how the local gene regulatory relationships may be related to COPD pathogenesis.

We found substantial partial correlation in gene expression between genes located in this cluster of COPD GWAS loci. Of interest, we identified potential biological connections between several of the COPD-related GWAS genes in this region, of which: *FAM13*-*TET2*, *BTC*-*NPNT* and *PPA2*-*HHIP* showed a consistently high partial correlation score. Furthermore, *BTC*, *NPNT*, *PPM1K* and *HHIP* appeared in the same network module in a consensus clustering approach, suggesting possible functional co-regulation. We also found these results robust to other phenotype controlling factors, cell composition (a notable source of bias in gene co-expression network analysis) and PPI randomization. We also observed intriguing differential co-expression between COPD cases versus controls. Multiple genes in this region have been discovered in previously performed cell-based knock-out experiments, including *SPP1* which strongly suggest the presence of a co-regulatory network in the chromosome 4q COPD risk region. These results will require functional validation, but these analyses provide a list of potential key gene expression regulators that may be related to COPD pathogenesis. Of interest, we also found significant partial correlations between regional COPD GWAS genes in another genomic region on chromosome 5q.

Some limitations of this study, and possible future works, include functional validation of the network relationships that we identified and further replication in different cohorts and possibly on different *-omics* data, although it was encouraging to see similar partial correlation signals in three independent cohorts. Also, it is worth nothing that we tried to overcome the uncertainty about what the key genes are in the COPD GWAS loci by using multiple sources of evidence, but we acknowledge that the evidence supporting some of the regional genes (e.g., *HHIP*, *FAM13A*) is stronger than others. Although the algorithm was developed to carry out whole genome analyses, in this manuscript we analyzed the transcriptomic data in a smaller region (224 genes). Future work could leverage the partial correlation algorithm across the whole genome where the number of genes is usually much greater than the number of samples. Furthermore, the analysis could be extended to DNA methylation profiles, protein expression levels, etc. The gene-specific weights used to marginalize the effect of surrounding genes when computing the partial correlation can leverage other biological sources other than the PPI.

In conclusion, while regional enrichment for GWAS signals may be a random event, the overall distribution of COPD GWAS loci across the genome is more clustered than expected by chance. The chromosome 4q region harbors some of the strongest genetic associations to COPD. Our results provide orthogonal evidence to COPD GWAS signals regarding the importance of this genomic region to COPD and suggest the possible existence of long-range gene regulatory mechanisms in this region.

## 4. Methods

### 4.1 Data

#### Chromosome 4q region (BTC-HHIP) selection

We downloaded the BioMart Ensembl database (https://www.ensembl.org/biomart/martview; accessed 06/25/2021). From the Human Ensembl Gene dataset we selected all genes, retrieving the gene position, and kept only genes with a transcriptional start site (bp) within the 4q COPD risk region, which covers 2Mb before the first top COPD GWAS SNP (rs2047409) and 2Mb after the fifth top COPD GWAS SNP (rs4585380), on the hg38 genome build. This process resulted in selecting a total of 280 genes.

#### Gene expression

In this study, we analyzed lung tissue samples from 458 COPD cases and 329 control subjects in the Lung Tissue Research Consortium (LTRC) [31]. Cases were spirometrically defined COPD patients with moderate to very severe COPD based on post-bronchodilator (or pre-bronchodilator if post-bronchodilator values were unavailable) spirometry (FEV1 < 80% predicted and FEV1/FVC < 0.7). Lung tissue samples with a pathologic diagnosis of Interstitial Lung Disease (ILD) were excluded from the analysis. mRNA sequencing was performed through the NHLBI TOPMed program at the University of Washington. RNA quantification was performed using the Quant-iT RNA assay (Invitrogen) and RNA integrity analysis was performed using a fragment analyzer (Advanced Analytical). Final RNASeq libraries were quantified using the Quant-it dsDNA High Sensitivity assay. Generation and bioinformatic processing of the libraries included: (1) generation of base calls in real-time on the NovaSeq6000 instrument (RTA 3.1.5); (2) demultiplexed, unaligned BAM files were produced by Picard ExtractIlluminaBarcodes and IlluminaBasecallsToSam and converted to FASTQ format using SamTools bam2fq (v1.4); (3) sequence read and base quality were checked using the FASTX-toolkit (v0.0.13); (4) Isoform-level expression quantification was generated with Salmon (v1.3.0) pseudoalignment to GENCODE release 37 transcriptome and summarized to gene-level TPM (transcripts per million) quantification using tximeta (v1.8.5). TPMs were scaled to obtain gene length corrected counts and we kept only genes with CPM (counts per million) above 0.5 in more than 50 samples. Upper-quartile normalization was applied to obtain log-CPM expression values that were further adjusted for library batch effects (limma v3.46.0 and edgeR v3.32.1). Gender consistency was confirmed by comparing the expression of Xist and non-pseudoautosomal regions of the Y chromosome. Finally, we removed genes that had log-CPM values > 0 in less than 75% of the patients, for a final count of 14,434 genes. There were 224 genes in the 4q COPD risk region.

#### Protein–Protein interaction network

We used the STRING PPI network [25] (https://string-db.org/cgi/download.pl; accessed 06/25/2021), a well-established protein-protein interactome, and selected only interactions identified using experimental evidence; this yielded a network consisting of 11,763 genes and 230,524 interactions.

### 4.2 Analytical methods

#### Clustering of GWAS signals

For each possible subsequent number of *k* hits (k = 1,2,3…) in each chromosome, we tested for the statistical significance (100,000 simulations) for *k* hits, out of the 83 COPD GWAS signals, to be present within their genome region length (*region_length*). In each simulation we sampled 83 loci at random across the genome and computed the number of regions with at least *k* hits within the *region_length*.

**Partial Correlation** computes the correlation between two genes while considering the indirect effects of all the other genes on that correlation. If we define the gene expression matrix as *X*∈*R*^*n*,*p*^, with n subjects and p observed genes, then the partial correlation between two genes g_i_ and g_j_ controlling for every other gene in *X*, is defined as the correlation of the residuals, r_i_ and r_j_, of the linear regressions with g_i_ and g_j_ as target variables and every other gene in *X* as regressing variables, *PCor*(g_i_, g_j_∣ *X*) = *Corr*(r_i_, r_j_),

#### High dimensional data partial correlation

Due to the high dimensionality of the data, we cannot compute the partial correlation score controlling for all other genes at the same time. Indeed, the ratio between the number of regressors (p), *i*.*e*., the number of genes in the genome (~20k), and the number of observations (n), *i*.*e*., the number of samples in the study (usually ~1k), is well above 1 (p>>n), which makes the calculation of the inverse matrix indeterminate.

In this work we introduce a gene-specific regularization factor when computing the Partial Correlation score to make the indeterminate regression feasible. Various attempts have been made to perform covariance selection for data sets with high dimensional gene expression data (p >> n) [32,33], assuming sparsity of the partial correlation matrix or introducing a regularization factor when computing the residuals on which to compute the partial correlation. Others have proposed a backward selection scheme to remove weak edges in the estimated precision matrix [34] or to estimate partial correlation using the Moore–Penrose pseudo-inverse of the covariance matrix [35]. Noting that the regression setting would allow sparsity to be more naturally incorporated in a penalized regression setting, we decided to move in this direction rather than computing the inverse of the covariance matrix.

Usually, in linear regression models the regularization factor is added in the optimization problem while minimizing the loss function. For the linear regression we can define:

$$\underset{\beta }{min}{\left\|y-X\beta \right\|}_{2}+\lambda \cdot L(\beta )$$ (1)

where *L*(*β*) is the regularization factor, usually a norm of the regression coefficients vector, among the most popular the Lasso, *L*(*β*) = |*β*|, and the Ridge, *L*(*β*) = ‖*β*‖_2_. It is worth noting that in this formulation, when computing the partial correlation, we control for every other gene in the same way; in other words, *λ* penalizes the whole vector *β* at the same time. In this paper, we suggest that the regularization parameter *λ* should be specific for each regression and for each controlling gene. Imagine that we are computing the partial correlation between two genes. We would like to condition this co-expression more for related genes, such as those in the same pathway or which are known to interact, than for genes that do not share any common functional activity or are very far apart in the protein-protein interaction network.

Consequently, we decided to slightly modify the computation of the residuals for the sparse partial correlation matrix, an approach we refer to as “Gene-Specific Ridge Regression”.

#### Definition of gene-specific ridge partial correlation

Given a gene expression Matrix X∈R^n,p^, and Protein-Protein interaction network, we define the gene-specific regularization vector λ_i,j_ as the inverse of the personalized PageRank score [36] towards the genes *i* and *j*. As a result, the partial correlation between two genes g_i_ and g_j_ is defined as:

$$PCorr(g_{i},g_{j})=Corr(r_{i,j},r_{j,i})$$ (2)

$$r_{i,j}=y_{i}-X{\hat{\beta }}_{i,j}$$ (3)

$${\hat{\beta }}_{i,j}=\underset{\beta }{argmin}{\left\|y_{i}-X\beta \right\|}_{2}+{\left\|\lambda_{i,j}∙\beta \right\|}_{2},$$ (4)

$$s.t.\beta_{i}=\beta_{j}=0$$ (5)

Where y_i_ is the expression of g_i_, *i*.*e*., the i-th column of *X*, β∈R^p^, and (*λ*_*i*,*j*_∙β) is defined as an element-wise vector product. The general pipeline to extract the partial correlation among genes is presented in **Fig 2**.

The main novelty is that in the regression for g_i_ and g_j_, we use a generalized ridge model and the regularization factor λ_i,j_ is a vector rather than a scalar. In the element-wise product (*λ*_*i*,*j*_∙β) each element $\lambda_{i,j}^{k}$ penalizes the coefficient of its corresponding gene (β_*k*_) individually. In this way, by using prior biological network information, such as the PPI, to define the values of the gene-specific regularization vector λ_i,j_, we can prioritize for the effect of other genes in the regression used in the computation of the partial correlation. The less g_k_ is related to g_i_ and g_j_ in the PPI, the higher the score $\lambda_{i,j}^{k}$ in the *λ*_*i*,*j*_ vector will be, thus the less g_k_ will be considered to regress the expression of g_i_. When g_k_ is less related to g_i_ and g_j_ in the PPI, the score $\lambda_{i,j}^{k}$ in the *λ*_*i*,*j*_ vector will be higher. As a result, g_k_ will have a higher penalization in the regression for g_i_.

#### Partial Correlation parameters

The above definition of the Partial Correlation allows us to tune the impact of the controlling genes on the partial correlation through two parameters: the magnitude of the gene-specific regularization vector *λ*_*i*,*j*_ (*min_lambda*) and the number of controlling genes (*n_genes)*, which have the lowest score in the *λ*_*i*,*j*_ vector. Depending on the way *λ*_*i*,*j*_ is computed from the prior biological network information, it can assume very high or low values which can drastically impact the Gene-Specific Ridge Partial Correlation. Thus, before plugging the *λ*_*i*,*j*_ into the Gene-Specific Ridge Regression, we normalize it so that its minimum value is equal to *min_lambda* (more details in **S1 Text**). Since there is not an optimization function to minimize and determine the optimal values for these two parameters, Section 2.2 provides results obtained by using different values *min_lambda* and *n_genes*.

#### Network edges

We analyzed both a COPD *specific network* (Section 2.3) and a *differential network* (Section 2.4). The COPD specific network represents relationships between genes in COPD subjects, including relationships that can be present in control subjects as well. The differential network is built to understand what relationships change between COPD and control subjects. To build these two networks, we computed two types of edges: significant edges and differential edges. The former tests whether a partial correlation between two genes is statistically significant within a reference population, e.g. COPD, while the latter tests whether the partial correlation is statistically different in two different populations, e.g., COPD Cases vs. Controls.

We assessed *significant edges* through a bootstrap approach with 1000 samplings, sampling with replacement from individuals in the Case and Control populations separately. The 1000 observations provide a distribution to test, using a one sample T-test for each edge. To increase the specificity of our network, *i*.*e*., edges that truly represent co-regulatory relationships, rather than testing the Partial Correlation to be greater than 0, we test each edge to be greater than the 95^th^ percentile (if positive) or smaller than the 5^th^ percentile (if negative) of the observed distribution for all the edges, which is roughly 0.15 and -0.15, respectively. Since this involved multiple testing of our hypothesis, we adjusted the p-value using the Benjamini/Hochberg (BH) procedure, and a threshold of 0.01 for false discovery rate.

To assess *differential edges* we used a permutation approach with 1000 samplings. More specifically, we shuffled case and control labels and computed the partial correlations for the new set of subjects. At each iteration, we computed the difference between the two permuted population’s scores. Through this procedure we test each edge with its own reference distribution (T-test) adjusting for multiple hypothesis testing (BH) and a threshold of 0.1 for false discovery rate. The less stringent threshold is due to the exploratory nature of the subsequent analyses allowing for the discovery of more differential edges that could be of biological interest.

#### PPI randomization

To further understand and elucidate the role of the PPI in the algorithm, we computed the partial correlation in two randomization settings: a randomized PPI and a randomized Prior. In the former case, we computed an expected degree randomization of the PPI, and then ran our partial correlation pipeline as usual. In the latter case, we computed the gene-specific PageRank matrix on the original PPI and then we randomized column-wise (controlling genes), so that the PageRank vector used for each gene (row of the PageRank matrix) would have the same distribution, but the controlling genes ({i,j}-entry of the PageRank Matrix) would each get a random regularization factor.

#### Network clustering

Network modules were computed using the Clauset-Newman-Moore greedy modularity maximization algorithm implemented in the *Networkx (*v2.8.2) Python library [37].

#### Cell-based knock-out functional enrichment analysis

We assessed functional effects of previously reported cell-based knock-out experiments through the *Enrichr* function of the *Gseapy* [38] *(v0*.*10*.*8)* Python library. Only enrichments from the LINCS L1000 project [29] CRISPR Knock-out with adjusted p-value < 0.01 were extracted.

**Modularity** is defined [39] as:

$$Q=\sum_{c}\left[\frac{L_{c}}{m}-{\left(\frac{k_{c}}{2m}\right)}^{2}\right]$$ (6)

where the sum iterates over all communities *c*, *m* is the number of edges in the network, *L*_*c*_ is the number of intra-community edges for community *c*, *k*_*c*_ are the degrees of the nodes in community *c*. Intuitively, this measure quantifies the density of connections within modules versus what would be expected given the degree distribution of the network. Modularity ranges from -1 to 1.

#### Co-clustering significance

To assess the significance of the co-clustering, we compared the number of times two genes appear in the same cluster to a Poisson distribution. The Poisson distribution represents the probability of a given number of events occurring in a fixed interval of time. In our case the event is to be in the same cluster. The *λ* parameter of the distribution, *i*.*e*., the expected rate of occurrences, represents the probability for two genes to appear in the same cluster with a random assignment.

## Supporting information

S1 Text Supporting information for the Partial Correlation algorithm. A detailed description of the Partial Correlation method used is included, and the choices made throughout the analysis pipeline are described.
(DOCX)

S1 Table Supportive Evidence for COPD Candidate Genes. The Variant to Gene **(**V2G) score of the *OpenTarget* platform [24] integrates evidence for genes within +/- 500kb from the selected GWAS variant, including: (1) Molecular phenotype quantitative trait loci experiments (QTLs), (2) Chromatin interaction experiments, e.g., Promoter Capture Hi-C (PCHi-C), (3) *In silico* functional predictions, e.g., Variant Effect Predictor (VEP) from Ensembl and (4) Distance between the variant and each gene’s canonical transcription start site (TSS)
(DOCX)

S2 Table Partial correlation values of COPD Candidate Genes (CCG). Minimum and Maximum partial correlation values of COPD Candidate Gene (CCG) nodes in different gene expression data sets. The distribution for each gene pair is obtained by the partial correlation values observed with different parameters of the algorithm.
(DOCX)

S3 Table Co-occurrence table of COPD Candidate Genes (*CCG)* in modules of the partial correlation networks. Co-occurrence table of *CCG* in modules of the partial correlation networks obtained by choosing different parameters. While *FAM13A* does not appear often in the same module with other CCG, most CCG co-occur in the same module with at least one other CCG. (*): statistically significant compared to a random cluster assignment (Poisson distribution), adjusted for multiple testing (FDR <0.05).
(DOCX)

S4 Table GSEA enrichment of the *HHIP NPNT BTC PPM1K* cluster. (DOCX)

S5 Table List of the 26 genomic regions that had at least three hits within 34.28 Mb. (DOCX)

S1 Fig Naïve example of Partial Correlation. Naïve example of Correlation vs Partial Correlation Networks. Assuming an unknown latent model that relates three variables, the Partial Correlation can remove indirect correlations between variables.
(TIF)

S2 Fig Boxplot of COPD Candidate Genes (*CCG)*. Boxplot of COPD Candidate Genes (*CCG)* partial correlation distribution over different parameter selections in the three GSE populations: GSE23352 (Laval), GSE23545 (Groningen), and GSE23529 (University of British Columbia). The demographic data are available through [26]. Interestingly, Groningen (GRNG) is the cohort with the most severe cases of COPD (lowest FEV1% predicted). A gene pair is identified by the position on the x-axis and the color of the distribution.
(TIF)

S3 Fig Partial correlations computed with and without phenotype correction. The two plots show the distribution of the absolute average difference between the partial correlations computed with and without phenotype correction for different parameters (*n_genes* and *min_lambda)*. For a fixed value of *n_genes* (x-axis), each boxplot includes the distribution of absolute average differences for all values of the *min_lambda* parameter, *i*.*e*. *[0*, *0*.*1*, *1*, *10]*. The left compares the partial correlation adding phenotype variables (age, sex, race, FEV1), the right plot compares the partial correlation model including the cell type proportion (epithelial, stromal, lymphoid).
(TIF)

S4 Fig Difference between the partial correlations computed with and without randomization. The distribution of the absolute average difference between the partial correlations computed with and without randomization for different parameters (*n_genes* and *min_lambda)* is shown. For a fixed value of *n_genes* (x-axis) the boxplot is the distribution over the different values of the *min_lambda* parameter, *i*.*e*. *[0*, *0*.*1*, *1*, *10]*. In orange is the randomized PPI setting, in blue is the randomized Prior. We computed the partial correlation in two randomization settings: a randomized PPI and a randomized Prior. In the former case, we computed an expected degree randomization of the PPI (as suggested in [27]), and then ran our partial correlation pipeline as usual. In the latter case, we computed the gene-specific PageRank matrix on the original PPI and then we randomized column-wise (controlling genes), so that the PageRank vector used for each gene (row of the PageRank matrix) would have the same distribution, but the controlling genes ({i,j}-entry of the PageRank Matrix) would each get a random regularization factor.
(TIF)

S5 Fig COPD GWAS hits plot. GWAS loci are the blue vertical lines. The small horizontal red lines identify 3 subsequent GWAS loci within a 34.28 MB window.
(TIF)

S6 Fig Chromosome 5 Partial Correlation network analysis. Partial Correlation network analysis results on Chromosome 5 COPD GWAS region. Left plot shows the partial correlation between CCG in this chromosomal region. Right plot shows the co-occurrence clustering of the genes in this region.
(TIF)
