# Supplementary material for: Partial correlation network analysis identifies coordinated gene expression within a regional cluster of COPD genome-wide association signals
Source: PLoS Comput Biol. 2024 Oct 17;20(10):e1011079. doi: 10.1371/journal.pcbi.1011079 (PMC11521246; doi:10.1371/journal.pcbi.1011079)
Supplement: S1 Text — A detailed description of the Partial Correlation method used is included, and the choices made throughout the analysis pipeline are described. (DOCX) [file pcbi.1011079.s001.docx]

## **Supplementary Material**

This Section contains a detailed description of the Partial Correlation method used and the choices made throughout the analysis pipeline. We also present further analyses that have been carried out to provide a more comprehensive understanding of the Partial Correlation Networks obtained. Furthermore, a python package has been developed and can be accessed at https://github.com/michelegentili93/Partial_Correlation

**Partial Correlation Algorithm Description**

To compute the Partial Correlation, it is required as input the Gene Expression Matrix (*X*), with *p* samples and *n* genes, a Protein-Protein interaction network (PPI), and two parameters (*n_genes,*$min\_lambda$*)*.

The algorithm runs in 3 steps:

- 1. Compute the Penalty Matrix between genes $\Lambda$
  2. Compute the Residual Matrix *R*
  3. Compute the Partial Correlation Matrix *PCORR*

**Step 1.** The Penalty Matrix is defined as a sequence of column vectors $\Omega=\left[ \omega_{i} \right]_{i=1}^{p}$, where each column vector $\omega_{i}$is the Personalized Page Rank [36] score computed on the PPI network with restart probability equal to 0.2, and teleporting the “*random walker*” exclusively to gene i ($g_{i})$.

**Step 2.** The partial correlation between two genes $g_{i}$ and $g_{j}$ is defined as:

$$\mathrm{PCorr}\left( g_{i},g_{j} \right)=Corr\left( r_{i,j},r_{j,i} \right)$$

$$r_{i,j}=y_{i}- X\hat{\beta}_{i,j}$$

$\hat{\beta}_{i,j}=\underset{\beta}{\mathrm{argmin}}{\|y_{i}-X\beta\|}_{2}+\left\| \lambda_{i,j}\cdot\beta\right\|_{2}$,

s.t. $\beta_{i}= \beta_{j}$ = 0 and $\beta_{k}=$ 0, $\forall k\notin top_{\lambda_{i,j}}$

Where $y_{i}$ is the expression of $g_{i}$, *i.e.,* the i-th column of *X,* $\beta\in R^{p}$, and $(\lambda_{i,j}\cdot\beta)$ is defined as an element-wise vector product. $\lambda_{i,j}$ is defined as the inverse of the combined personalized PageRank score of $g_{i}$ and $g_{j}$, $\lambda_{i,j}=\frac{1}{\omega_{i}+\omega_{j}}$*.* Intuitively, the higher $\left( \omega_{i}+\omega_{j} \right)_{k}$, the more relevant $g_{k}$ is for $g_{i}$ and $g_{j}$ in the PPI, and so the smaller the penalty score $\lambda_{i,j}^{k}$, used in the Ridge Regression. We finally normalize the column vector $\lambda_{i,j}=\frac{\lambda_{i}}{min\left( \lambda_{i} \right)}*min\_lambda$, so that the smallest penalization factor is equal to $min\_lambda$. If $g_{i}$ is not present in the PPI then $\omega_{i}=[\frac{1}{n},\frac{1}{n},\ldots,\frac{1}{n}$]. The top *n_genes* in$\lambda_{i,j}$ will form the set $top_{\lambda_{i,j}},$so that to compute the residuals of $g_{i} \mathrm{and}g_{j}$ we will use only the top *n_genes* closest in the PPI.

**Step 3.** Finally, the Partial Correlation between two genes is computed as the correlation of their residuals.

**Partial Correlation Network construction**

In brief the partial correlation network is constructed by computing the partial correlation on the gene expression profiles of the population of interest (COPD cases in our manuscript). We run a bootstrap approach to obtain a p-value for each edge. We set threshold of 0.01 for false discovery rate and finally we build a network with the edges that are significant.

The differential partial correlation network is obtained by computing the difference of the partial correlations obtained on the two populations’ gene expression profiles. The significance is obtained with a randomization approach (n=1000), in each iteration we randomized the phenotype of interest, and we compute again the difference of the partial correlation scores. This procedure provides a distribution against which we can test the significance of the differential partial correlation observed.

**Estimation of Partial Correlation Networks in LTRC data:**

Data inputs:

1. Processed RNA-seq data for 224 genes in the 4q COPD risk region in 458 COPD cases and 329 controls (processing described in Section 4.1).
2. PPI as a Boolean adjacency matrix from STRINGDB (processed as described in Section 4.1), 11,763 genes and 230,524 interactions.
3. Choose parameters *n_genes* = [0, 1, 5, 10, 25, 50, 75, 100], *min_lambda* = [0, 0.1, 1, 10]

Network estimation:

1. Partial correlation network:

For each set of parameters, we draw an edge between gene i and gene j if they have a statistically significant partial correlation (FDR <0.01) based on the following procedure:

- - 1. Through sampling (n=1000) with replacement (Section 4.2 *Network edges),* estimate distribution of partial correlations for each gene pair (edge).
    2. Using a one-sided t-test, compute p-value for the absolute partial correlation distribution mean being greater than threshold (0.15).
    3. Compute FDR on p-values from all gene pairs.

1. Differential partial correlation network:
   1. For each set of parameters, estimate Gene Specific Partial Ridge Regression (see Section 2.2 and 2.3 for details) in COPD cases and controls separately.
   2. For each gene pair (edge):
      1. compute the difference between COPD cases and controls.
      2. Through random label shuffling (COPD cases and controls) (Section 4.2 *Network edges)*, compute reference distribution for each gene pair (edge), compute p-value and threshold FDR <0.1

**Algorithm to build a partial correlation network**

**Input**:

GE = gene expression (*n* subjects, *p* genes)

gene_list = list of *p* genes

ml,ng = min_lambda, n_genes (PCorr algorithm parameters)

Pcorr_threshold = 0.15

adj_pvalue_threshold = 0.01

**Functions:**

Pcorr(g_i_,g_j_) = Partial Correlation Algorithm between gene i and gene j. Defined above in the **Partial Correlation Algorithm Description** subsection**.**

ttest() = One-sided t-test

Multitest_correction() = multiple hypothesis testing, Benjamini/Hochberg procedure

Sample(GE,n) = Sample *n* subjects with replacement from GE data set

**Procedure**:

Pvalue = list()

For each (g_i_,g_j_) pair in gene_list:

Pcorr_iter = list()

For iter in range(1000):

*# In each iteration sample n subjects with replacement from GE data set*

GE_sample = Sample(GE,n)

exp_g_i_, exp_g_j_ = extract gene i and gene j expressions from GE_sample

*# Compute and store the partial correlation for this pair of genes and this iteration*

Pcorr_iter[iter] = Pcorr(exp_g_i_, exp_g_j_)

*# Compute the statistical test for the partial correlation to be greater than threshold of 0.15.*

Pvalue[(g_i_,g_j_)] = ttest(abs(Pcorr_iter), Pcorr_threshold)

*# Compute adjusted pvalue with FDR correction*

Adj_Pvalue = Multitest_correction(Pvalue)

*# Extract the edges that are significant at FDR<0.01*

Significant_network = {edge_(gi,gj) if Adj_pvalue[(gi,gj)]< adj_pvalue_threshold for each (gi,gj) pair}

**Output:** Significant_network

**Algorithm to build a Differential partial correlation network**

**Input**:

GE = gene expression (*n* subjects, *p* genes)

Pheno_vector = Boolean vector for *n* subjects, 1 is case and 0 is control

gene_list = list of genes

ml,ng = min_lambda, n_genes (PCorr algorithm parameters)

Pcorr_threshold = 0.15

adj_pvalue_threshold = 0.1

**Functions:**

Pcorr(g_i_,g_j_) = Partial Correlation Algorithm between gene i and gene j. Defined above in the **Partial Correlation Algorithm Description** subsection.

ttest() = One sample two-sided t-test

Multitest_correction() = multiple hypothesis testing, Benjamini/Hochberg procedure

Randomize(vector) = Randomly shuffle the entries of the vector

**Procedure**:

Pvalue = list()

For each (g_i_,g_j_) pair in gene_list:

*# Compute Partial Correlations in Cases*

Pcorr_1 =_ Pcorr(g_i_[Pheno_vector==1], g_j_[Pheno_vector==1])

*# Compute Partial Correlations in Controls*

Pcorr_0 =_ Pcorr(g_i_[Pheno_vector==0], g_j_[Pheno_vector==0])

*# Compute Difference between Cases and Controls Partial Correlation*

Diff_obs = Pcorr_1_ - Pcorr_0_

Diff_iter = list()

For iter in range(1000):

# *Randomize entry of Pheno_vector*

Pheno_rnd = Randomize(Pheno_vector)

*# Compute Partial Correlations in Cases*

Pcorr_1 =_ Pcorr(g_i_[Pheno_rnd ==1], g_j_[Pheno_rnd ==1])

*# Compute Partial Correlations in Controls*

Pcorr_0 =_ Pcorr(g_i_[Pheno_rnd ==0], g_j_[Pheno_rnd ==0])

*# Compute and store Difference between Cases and Controls Partial Correlation*

Diff_iter[iter] = Pcorr_1_ - Pcorr_0_

Diff_mean = mean(Diff_iter)

Std_mean = std(Diff_iter)

*# Compute statistical significance of the Partial Correlation difference*

Pvalue[(g_i_,g_j_)] = 2 * (1 – normal_cdf(Diff_obs, loc= Diff_mean, scale = Std_mean))

*# Compute adjusted pvalue with FDR correction*

Adj_Pvalue = Multitest_correction(Pvalue)

*# Extract the edges that are significant, FDR<0.01*

Differential_network = {edge_(gi,gj) if Adj_pvalue[(gi,gj)]< adj_pvalue_threshold for each (gi,gj) pair}

**Output:** Differential_network
