# Supplementary material for: Partial correlation network analysis identifies coordinated gene expression within a regional cluster of COPD genome-wide association signals
Source: PLoS Comput Biol. 2024 Oct 17;20(10):e1011079. doi: 10.1371/journal.pcbi.1011079 (PMC11521246; doi:10.1371/journal.pcbi.1011079)
Supplement: S1 Table — The Variant to Gene (V2G) score of the OpenTarget platform [24] integrates evidence for genes within +/- 500kb from the selected GWAS variant, including: (1) Molecular phenotype quantitative trait loci experiments (QTLs), (2) Chromatin interaction experiments, e.g., Promoter Capture Hi-C (PCHi-C), (3) In silico functional predictions, e.g., Variant Effect Predictor (VEP) from Ensembl and (4) Distance between the variant and each gene’s canonical transcription start site (TSS) (DOCX) [file pcbi.1011079.s002.docx]

**S1 Table:** Supportive Evidence for COPD Candidate Genes. The Variant to Gene **(**V2G) score of the *OpenTarget* platform[25] integrates evidence for genes within +/- 500kb from the selected GWAS variant, including: (1) Molecular phenotype quantitative trait loci experiments (QTLs), (2) Chromatin interaction experiments, e.g., Promoter Capture Hi-C (PCHi-C), (3) *In silico* functional predictions, e.g., Variant Effect Predictor (VEP) from Ensembl and (4) Distance between the variant and each gene's canonical transcription start site (TSS)

| **GWAS SNP** | **Candidate Gene** | **Variant to Gene Ranking in OpenTargets** | **Evidence from COPD GWAS Bioinformatic Analysis (Sakornsakolpat 2019)**** |
| --- | --- | --- | --- |
| rs4585380 | *BTC* | 1st | None |
| rs4585380 | *EPGN* | >3rd | Hi-C,DHS |
| rs7671261 | *FAM13A* | 1st | GREx, mQTL, DHS |
| rs7671261 | *NAP1L5* | 3rd | mQTL, Hi-C |
| rs7671261 | *PPM1K* | N/A^*^ | Cod., DHS |
| rs34712979 | *NPNT* | 1st | DHS, Gset |
| rs34712979 | *PPA2* | >3rd | GREx, DHS |
| rs13140176 | *HHIP* | 1st | GREx, DHS, GSet |
| rs2047409 | *TET2* | 1st | None |

* *PPM1K* is >500kb away from rs7671261, thus doesn’t have a V2G score

** Abbreviations:

GREx: Genetically regulated gene expression (S-PrediXcan)

DHS: DNase hypersensitivity sites

GSet: Target genes identified by DEPICT using reconstituted gene sets

Cod: Significant single variant or gene-based association tests for deleterious coding variants

Hi-C: Significant chromatin interaction identified in human lung or IMR90 cell line

mQTL: Colocalization of methylation quantitative trait locus and GWAS signals with posterior probability >0.1
