# Supplementary material for: Partial correlation network analysis identifies coordinated gene expression within a regional cluster of COPD genome-wide association signals
Source: PLoS Comput Biol. 2024 Oct 17;20(10):e1011079. doi: 10.1371/journal.pcbi.1011079 (PMC11521246; doi:10.1371/journal.pcbi.1011079)
Supplement: S2 Table — Minimum and Maximum partial correlation values of COPD Candidate Gene (CCG) nodes in different gene expression data sets. The distribution for each gene pair is obtained by the partial correlation values observed with different parameters of the algorithm. (DOCX) [file pcbi.1011079.s003.docx]

**S2 Table**: **Partial correlation values of COPD Candidate Gene (CCG).**

Minimum and Maximum partial correlation values of COPD Candidate Gene (CCG) nodes in different gene expression data sets. The distribution for each gene pair is obtained by the partial correlation values observed with different parameters of the algorithm.

| **edge** | **LTRC case** | **LTRC control** | **GSE23352** | **GSE23529** | **GSE23545** |
| --- | --- | --- | --- | --- | --- |
| **BTC-FAM13A** | -0.0 [-0.03, 0.17] | 0.01 [-0.03, 0.27] | -0.0 [-0.01, 0.04] | -0.05 [-0.1, -0.02] | -0.02 [-0.1, 0.02] |
| **BTC-HHIP** | -0.09 [-0.1, -0.08] | -0.11 [-0.12, -0.08] | 0.05 [0.03, 0.06] | -0.01 [-0.03, 0.01] | -0.06 [-0.13, -0.04] |
| **BTC-NAP1L5** | -0.05 [-0.07, 0.02] | -0.01 [-0.03, 0.03] | -0.05 [-0.07, -0.03] | -0.02 [-0.06, 0.02] | -0.06 [-0.09, -0.02] |
| **BTC-NPNT** | 0.27 [0.26, 0.29] | 0.17 [0.16, 0.23] | 0.17 [0.15, 0.27] | 0.18 [0.13, 0.29] | 0.27 [0.26, 0.3] |
| **BTC-PPA2** | -0.04 [-0.07, 0.03] | -0.12 [-0.19, -0.1] | -0.02 [-0.06, 0.01] | -0.18 [-0.21, -0.14] | -0.1 [-0.15, -0.07] |
| **BTC-PPM1K** | -0.02 [-0.09, 0.01] | 0.06 [-0.01, 0.07] | -0.02 [-0.06, -0.01] | -0.03 [-0.04, -0.01] | -0.2 [-0.22, -0.18] |
| **BTC-TET2** | 0.02 [-0.01, 0.07] | -0.06 [-0.12, 0.0] | -0.07 [-0.11, -0.04] | -0.08 [-0.1, -0.07] | -0.05 [-0.09, 0.03] |
| **FAM13A-HHIP** | -0.05 [-0.06, -0.04] | -0.03 [-0.04, 0.03] | 0.05 [0.01, 0.11] | 0.07 [0.03, 0.13] | 0.01 [-0.03, 0.13] |
| **FAM13A-NAP1L5** | 0.03 [-0.05, 0.08] | 0.11 [0.09, 0.15] | 0.04 [-0.03, 0.06] | -0.01 [-0.05, 0.03] | 0.0 [-0.01, 0.02] |
| **FAM13A-NPNT** | -0.04 [-0.05, -0.03] | -0.03 [-0.05, 0.01] | 0.09 [0.04, 0.2] | 0.1 [0.05, 0.15] | -0.05 [-0.07, 0.03] |
| **FAM13A-PPA2** | -0.03 [-0.06, -0.0] | -0.02 [-0.07, -0.01] | -0.09 [-0.1, -0.08] | 0.02 [-0.01, 0.05] | 0.09 [0.05, 0.18] |
| **FAM13A-PPM1K** | 0.04 [-0.01, 0.08] | 0.04 [0.02, 0.19] | 0.05 [0.03, 0.09] | -0.01 [-0.04, 0.06] | 0.04 [0.01, 0.11] |
| **FAM13A-TET2** | 0.18 [0.11, 0.22] | 0.07 [0.05, 0.17] | 0.06 [0.03, 0.08] | 0.11 [0.08, 0.12] | 0.15 [0.08, 0.21] |
| **HHIP-NAP1L5** | 0.02 [0.0, 0.04] | 0.08 [0.04, 0.1] | 0.07 [0.05, 0.07] | 0.01 [-0.01, 0.03] | 0.02 [-0.06, 0.03] |
| **HHIP-NPNT** | 0.2 [0.07, 0.27] | 0.19 [0.07, 0.29] | 0.21 [0.08, 0.25] | 0.18 [0.02, 0.23] | 0.21 [0.07, 0.25] |
| **HHIP-PPA2** | 0.2 [0.13, 0.22] | 0.18 [0.15, 0.18] | 0.11 [0.07, 0.12] | 0.02 [-0.0, 0.06] | -0.04 [-0.08, -0.03] |
| **HHIP-PPM1K** | 0.19 [-0.03, 0.22] | 0.18 [0.05, 0.26] | 0.05 [0.04, 0.07] | -0.04 [-0.06, -0.01] | -0.03 [-0.05, -0.0] |
| **HHIP-TET2** | -0.01 [-0.03, 0.01] | 0.01 [0.01, 0.03] | 0.09 [0.03, 0.12] | -0.02 [-0.04, 0.0] | -0.06 [-0.09, -0.04] |
| **NAP1L5-NPNT** | -0.03 [-0.06, -0.01] | 0.01 [-0.01, 0.03] | -0.02 [-0.05, 0.01] | -0.01 [-0.03, 0.06] | -0.08 [-0.12, -0.06] |
| **NAP1L5-PPA2** | -0.04 [-0.06, -0.02] | 0.0 [-0.05, 0.1] | -0.01 [-0.05, 0.02] | -0.09 [-0.11, -0.06] | 0.06 [0.03, 0.08] |
| **NAP1L5-PPM1K** | 0.0 [-0.01, 0.02] | -0.04 [-0.05, -0.01] | -0.0 [-0.03, 0.03] | -0.06 [-0.07, -0.02] | 0.05 [0.02, 0.11] |
| **NAP1L5-TET2** | 0.04 [-0.03, 0.08] | -0.03 [-0.12, 0.01] | -0.0 [-0.01, 0.05] | 0.06 [-0.01, 0.07] | 0.03 [-0.02, 0.07] |
| **NPNT-PPA2** | 0.0 [0.0, 0.02] | -0.12 [-0.14, -0.09] | -0.01 [-0.03, 0.03] | -0.15 [-0.17, -0.14] | -0.12 [-0.2, -0.09] |
| **NPNT-PPM1K** | 0.23 [0.09, 0.33] | 0.17 [0.02, 0.35] | 0.18 [0.08, 0.24] | -0.0 [-0.02, -0.0] | 0.06 [-0.07, 0.08] |
| **NPNT-TET2** | -0.14 [-0.15, -0.09] | -0.09 [-0.14, -0.08] | 0.08 [-0.06, 0.14] | -0.18 [-0.21, -0.14] | 0.09 [-0.0, 0.1] |
| **PPA2-PPM1K** | -0.1 [-0.16, -0.04] | -0.22 [-0.3, -0.0] | -0.11 [-0.17, -0.07] | 0.05 [0.0, 0.08] | -0.01 [-0.02, 0.05] |
| **PPA2-TET2** | -0.14 [-0.27, -0.01] | -0.27 [-0.45, -0.11] | 0.28 [0.27, 0.33] | 0.42 [0.37, 0.45] | 0.07 [0.06, 0.08] |
| **PPM1K-TET2** | 0.27 [0.15, 0.33] | 0.29 [0.23, 0.37] | 0.03 [0.0, 0.12] | -0.13 [-0.14, -0.12] | -0.05 [-0.06, -0.02] |
