# Supplementary material for: Partial correlation network analysis identifies coordinated gene expression within a regional cluster of COPD genome-wide association signals
Source: PLoS Comput Biol. 2024 Oct 17;20(10):e1011079. doi: 10.1371/journal.pcbi.1011079 (PMC11521246; doi:10.1371/journal.pcbi.1011079)
Supplement: S3 Table — Co-occurrence table of CCG in modules of the partial correlation networks obtained by choosing different parameters. While FAM13A does not appear often in the same module with other CCG, most CCG co-occur in the same module with at least one other CCG. (*): statistically significant compared to a random cluster assignment (Poisson distribution), adjusted for multiple testing (FDR <0.05). (DOCX) [file pcbi.1011079.s004.docx]

**S3 Table: Co-occurrence table of COPD Candidate Genes (*CCG)* in modules of the partial correlation networks.**  Co-occurrence table of *CCG* in modules of the partial correlation networks obtained by choosing different parameters. While *FAM13A* does not appear often in the same module with other CCG, most CCG co-occur in the same module with at least one other CCG. (*): statistically significant compared to a random cluster assignment (Poisson distribution), adjusted for multiple testing (FDR <0.05).

|  | **FAM13A** | **HHIP** | **PPA2** | **BTC** | **TET2** | **NPNT** | **PPM1K** |
| --- | --- | --- | --- | --- | --- | --- | --- |
| **FAM13A** | 0 | 0 | 2 | 0 | 4 | 1 | 3 |
| **HHIP** | 0 | 0 | 3 | 16* | 4 | 15* | 10 |
| **PPA2** | 2 | 3 | 0 | 3 | 11 | 3 | 8 |
| **BTC** | 0 | 16* | 3 | 0 | 9 | 23* | 8 |
| **TET2** | 4 | 4 | 11 | 9 | 0 | 10 | 4 |
| **NPNT** | 1 | 15* | 3 | 23* | 10 | 0 | 9 |
| **PPM1K** | 3 | 10 | 8 | 8 | 4 | 9 | 0 |
