# Supplementary material for: Partial correlation network analysis identifies coordinated gene expression within a regional cluster of COPD genome-wide association signals
Source: PLoS Comput Biol. 2024 Oct 17;20(10):e1011079. doi: 10.1371/journal.pcbi.1011079 (PMC11521246; doi:10.1371/journal.pcbi.1011079)
Supplement: S4 Table — (DOCX) [file pcbi.1011079.s005.docx]

**S4 Table:** GSEA enrichment of the *HHIP* *NPNT* *BTC* *PPM1K* cluster

| Gene_set | Term | Adjusted P-value | CCG |
| --- | --- | --- | --- |
| ENCODE Histone Modifications 2015 | H3K9me3 CD14-positive monocyte hg19 | 5.15E-04 | HHIP NPNT BTC |
| ENCODE Histone Modifications 2015 | H3K27me3 fibroblast of lung hg19 | 5.15E-04 | HHIP NPNT BTC |
| ENCODE Histone Modifications 2015 | H3K27me3 A549 hg19 | 9.33E-04 | PPM1K BTC |
| ENCODE Histone Modifications 2015 | H3K27me3 GM12878 hg19 | 9.33E-04 | HHIP NPNT BTC |
| ENCODE Histone Modifications 2015 | H4K20me1 A549 hg19 | 9.33E-04 | HHIP PPM1K |
| Epigenomics Roadmap HM ChIP-seq | H3K27me3 CD4+ CD25- CD45RA+ Naive Primary Cells | 1.11E-03 | HHIP BTC |
| Epigenomics Roadmap HM ChIP-seq | H3K27me3 Mobilized CD34 Primary Cells | 1.11E-03 | HHIP NPNT BTC |
| ENCODE Histone Modifications 2015 | H3K27me3 kidney epithelial cell hg19 | 1.18E-03 | NPNT |
| Epigenomics Roadmap HM ChIP-seq | H3K27me3 Brain Substantia Nigra | 1.35E-03 | BTC NPNT |
| Epigenomics Roadmap HM ChIP-seq | H3K27me3 CD4 Naive Primary Cells | 1.35E-03 | NPNT |
