# Supplementary material for: Partial correlation network analysis identifies coordinated gene expression within a regional cluster of COPD genome-wide association signals
Source: PLoS Comput Biol. 2024 Oct 17;20(10):e1011079. doi: 10.1371/journal.pcbi.1011079 (PMC11521246; doi:10.1371/journal.pcbi.1011079)
Supplement: S5 Table — (DOCX) [file pcbi.1011079.s006.docx]

**S5 Table:** List of the 26 genomic regions that had at least three hits within 34 Mb

| **Chr** | **Rs_id_1** | **Rs_id_2** | **Rs_id_3** |
| --- | --- | --- | --- |
| **1** | rs9435731 | rs76841360 | rs4660861 |
| **1** | rs76841360 | rs4660861 | rs72673419 |
| **1** | rs3009947 | rs11118406 | rs11579382 |
| **2** | rs955277 | rs10929386 | rs12466981 |
| **2** | rs2571445 | rs16825267 | rs62191105 |
| **3** | rs2442776 | rs1529672 | rs13073544 |
| **3** | rs1529672 | rs13073544 | rs17759204 |
| **3** | rs13073544 | rs17759204 | rs62259026 |
| **3** | rs4093840 | rs2955083 | rs7650602 |
| **4** | rs4585380 | rs7671261 | rs2047409 |
| **4** | rs7671261 | rs2047409 | rs34712979 |
| **5** | rs62375246 | rs10037493 | rs979453 |
| **5** | rs10037493 | rs979453 | rs10866659 |
| **5** | rs979453 | rs10866659 | rs12519165 |
| **6** | rs1334576 | rs9350191 | rs13198656 |
| **6** | rs9350191 | rs13198656 | rs3095329 |
| **6** | rs13198656 | rs3095329 | rs2070600 |
| **6** | rs2806356 | rs1631199 | rs646695 |
| **6** | rs1631199 | rs646695 | rs9399401 |
| **9** | rs7866939 | rs10760580 | rs803923 |
| **10** | rs2579762 | rs721917 | rs1570221 |
| **15** | rs72731149 | rs1441358 | rs55676755 |
| **15** | rs1441358 | rs55676755 | rs10152300 |
| **17** | rs8080772 | rs34727469 | rs62065216 |
| **17** | rs34727469 | rs62065216 | rs12185268 |
| **17** | rs62065216 | rs12185268 | rs11655567 |
