# Supplementary figures and images for: Partial correlation network analysis identifies coordinated gene expression within a regional cluster of COPD genome-wide association signals
Source: PLoS Comput Biol. 2024 Oct 17;20(10):e1011079. doi: 10.1371/journal.pcbi.1011079 (PMC11521246; doi:10.1371/journal.pcbi.1011079)

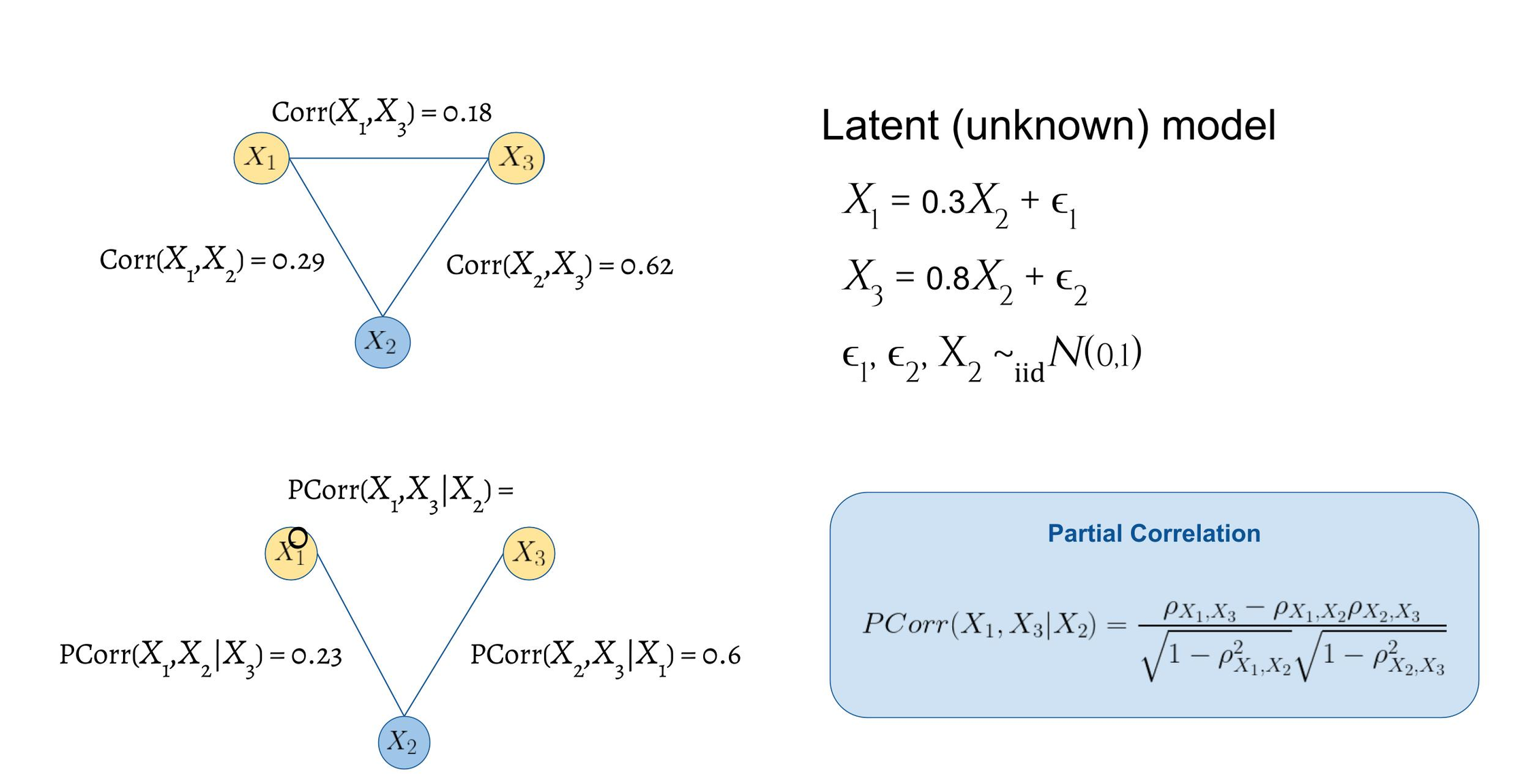

Supplement: S1 Fig — Naïve example of Correlation vs Partial Correlation Networks. Assuming an unknown latent model that relates three variables, the Partial Correlation can remove indirect correlations between variables. (TIF) [file pcbi.1011079.s007.tif]

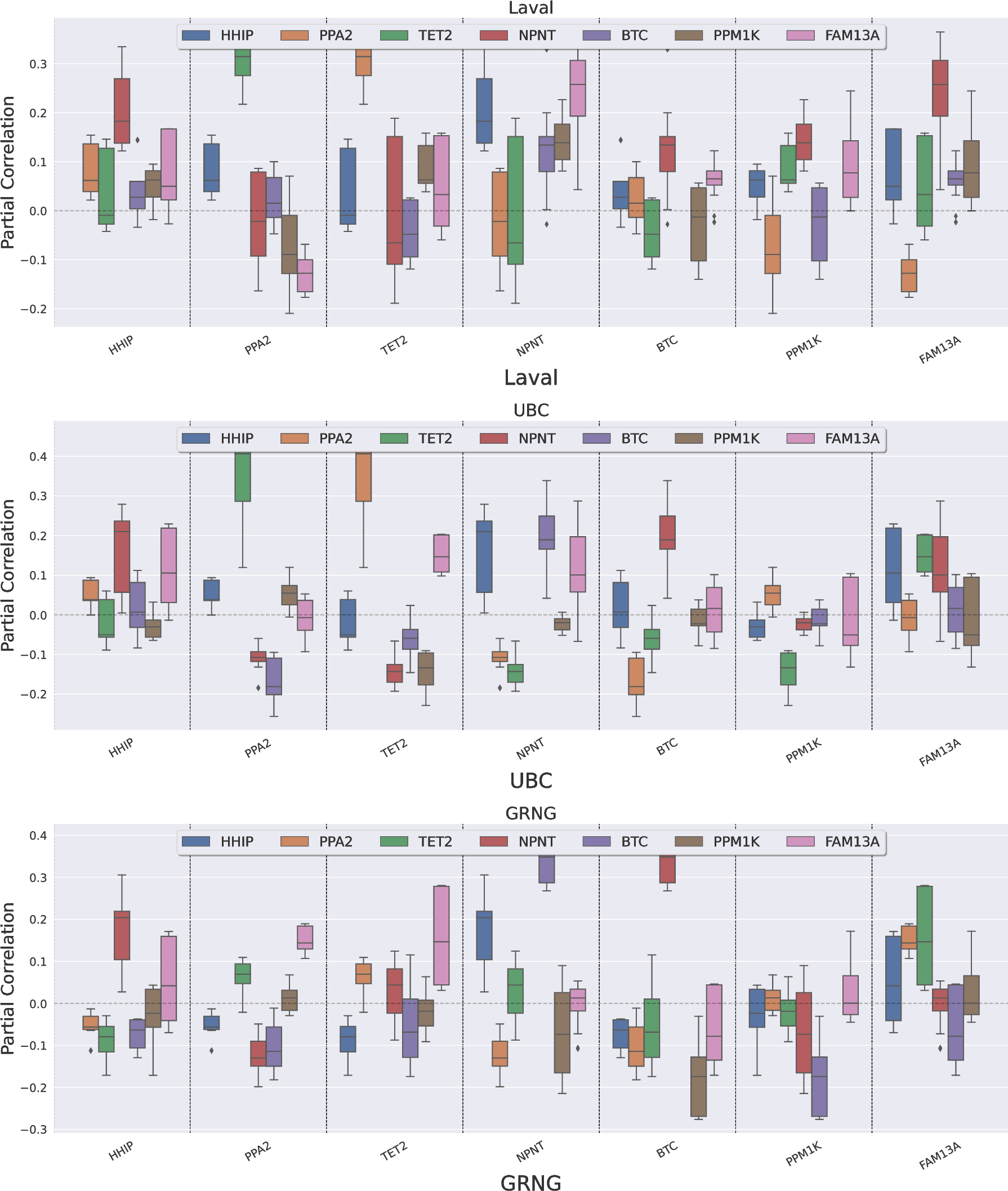

Supplement: S2 Fig — Boxplot of COPD Candidate Genes (CCG) partial correlation distribution over different parameter selections in the three GSE populations: GSE23352 (Laval), GSE23545 (Groningen), and GSE23529 (University of British Columbia). The demographic data are available through [26]. Interestingly, Groningen (GRNG) is the cohort with the most severe cases of COPD (lowest FEV1% predicted). A gene pair is identified by the position on the x-axis and the color of the distribution. (TIF) [file pcbi.1011079.s008.tif]

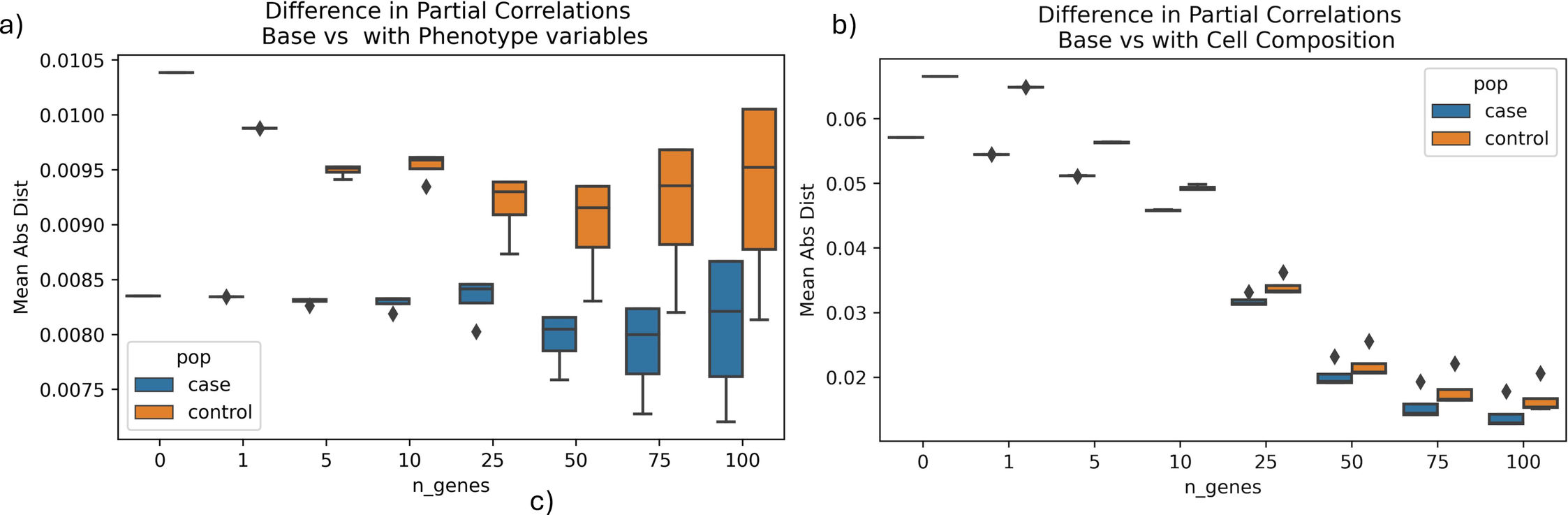

Supplement: S3 Fig — The two plots show the distribution of the absolute average difference between the partial correlations computed with and without phenotype correction for different parameters (n_genes and min_lambda). For a fixed value of n_genes (x-axis), each boxplot includes the distribution of absolute average differences for all values of the min_lambda parameter, i.e. [0, 0.1, 1, 10]. The left compares the partial correlation adding phenotype variables (age, sex, race, FEV1), the right plot compares the partial correlation model including the cell type proportion (epithelial, stromal, lymphoid). (TIF) [file pcbi.1011079.s009.tif]

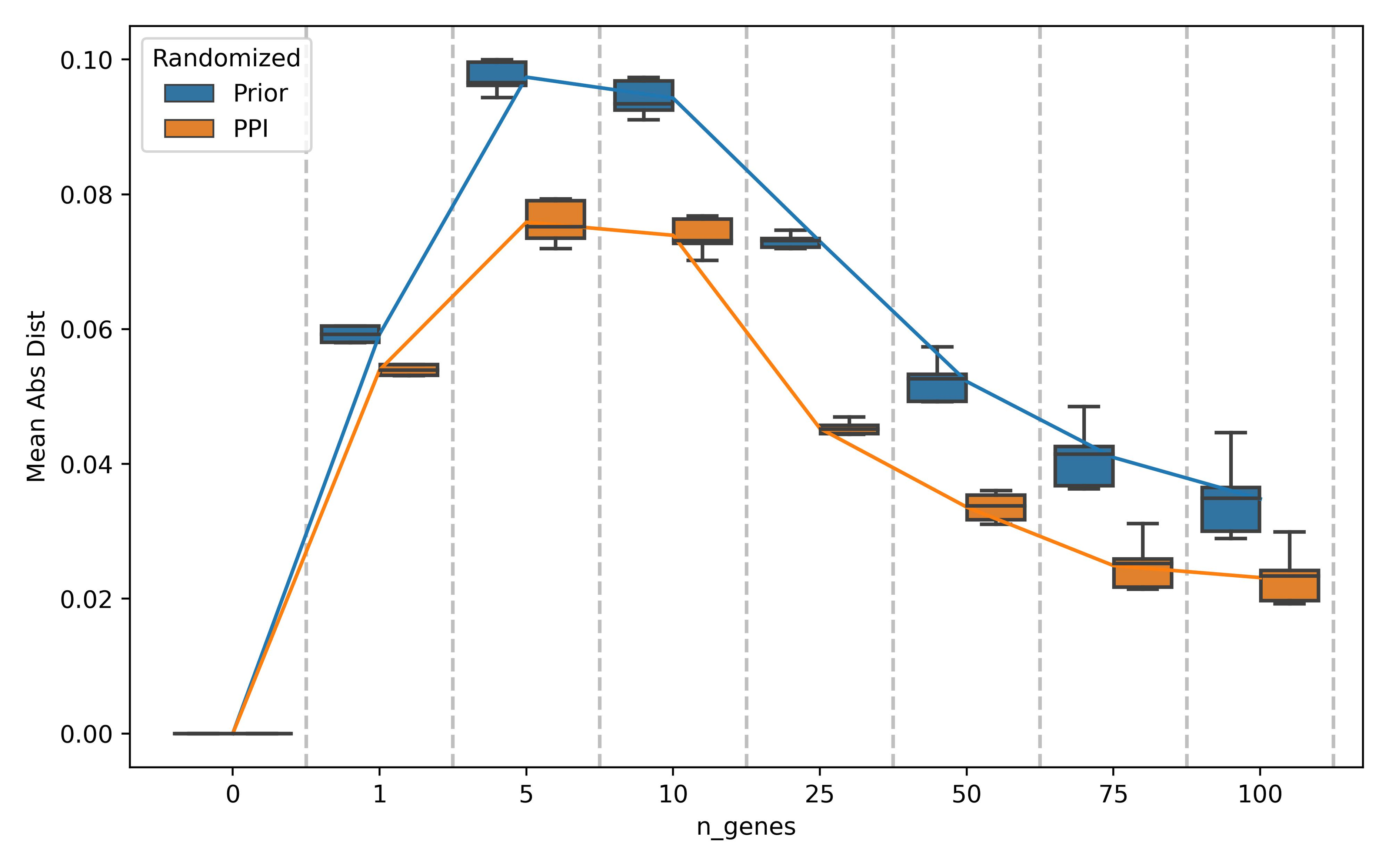

Supplement: S4 Fig — The distribution of the absolute average difference between the partial correlations computed with and without randomization for different parameters (n_genes and min_lambda) is shown. For a fixed value of n_genes (x-axis) the boxplot is the distribution over the different values of the min_lambda parameter, i.e. [0, 0.1, 1, 10]. In orange is the randomized PPI setting, in blue is the randomized Prior. We computed the partial correlation in two randomization settings: a randomized PPI and a randomized Prior. In the former case, we computed an expected degree randomization of the PPI (as suggested in [27]), and then ran our partial correlation pipeline as usual. In the latter case, we computed the gene-specific PageRank matrix on the original PPI and then we randomized column-wise (controlling genes), so that the PageRank vector used for each gene (row of the PageRank matrix) would have the same distribution, but the controlling genes ({i,j}-entry of the PageRank Matrix) would each get a random regularization factor. (TIF) [file pcbi.1011079.s010.tif]

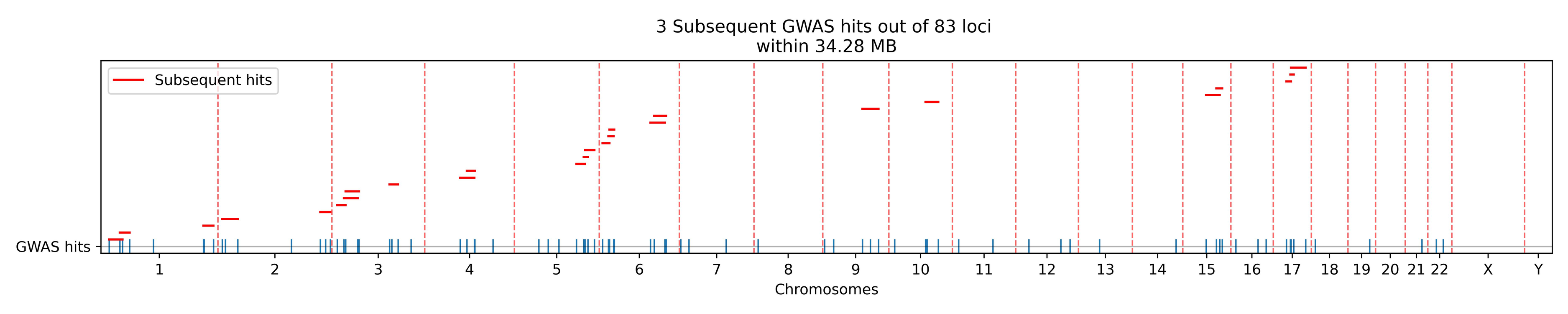

Supplement: S5 Fig — GWAS loci are the blue vertical lines. The small horizontal red lines identify 3 subsequent GWAS loci within a 34.28 MB window. (TIF) [file pcbi.1011079.s011.tif]

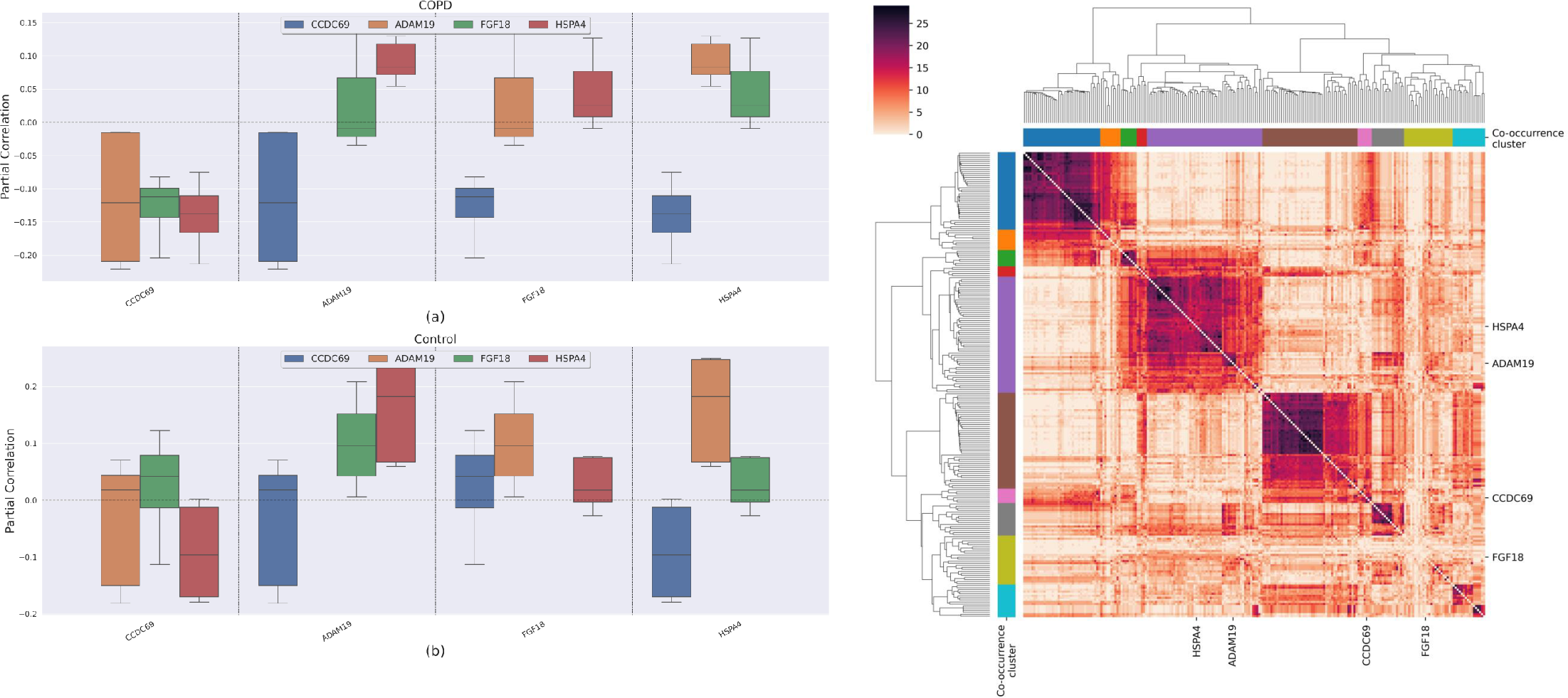

Supplement: S6 Fig — Partial Correlation network analysis results on Chromosome 5 COPD GWAS region. Left plot shows the partial correlation between CCG in this chromosomal region. Right plot shows the co-occurrence clustering of the genes in this region. (TIF) [file pcbi.1011079.s012.tif]
